# p97 Inhibitors Possessing Antiviral Activity Against SARS-CoV-2 and Low Cytotoxicity

**DOI:** 10.3390/ph18010131

**Published:** 2025-01-19

**Authors:** Rui Ding, Tiffany C. Edwards, Prithwish Goswami, Daniel J. Wilson, Christine D. Dreis, Yihong Ye, Robert J. Geraghty, Liqiang Chen

**Affiliations:** 1 Center for Drug Design, College of Pharmacy, University of Minnesota, Minneapolis, MN 55455, USAgoswamip@umn.edu (P.G.); dreis020@umn.edu (C.D.D.); 2 Laboratory of Molecular Biology, National Institute of Diabetes and Digestive and Kidney Diseases, National Institutes of Health, Bethesda, MD 20892, USA; yihongy@niddk.nih.gov

**Keywords:** p97, p97 inhibitor, antiviral, SARS-CoV-2, coronavirus

## Abstract

**Background:** p97 (also known as valosin-containing protein, VCP) is a member of the AAA+ ATPase family and is intimately associated with protein quality control and homeostasis regulation. Therefore, pharmaceutical inhibition of p97 has been actively pursued as an anticancer strategy. Recently, p97 has emerged as an important pro-viral host factor and p97 inhibitors are being evaluated as potential antiviral agents. **Methods:** We designed and synthesized novel p97 inhibitors based on the rearrangement of the central fused ring of our previously reported p97 inhibitors. These compounds were tested for inhibition of p97, cytotoxicity, and antiviral activity against SARS-CoV-2. Molecular docking was also performed on selected inhibitors to shed light on their binding modes. **Results:** Among these new p97 inhibitors, two compounds possess enhanced anti-p97 activity over their parent compounds. More significantly, these two inhibitors exhibit strong antiviral activity against SARS-CoV-2 at doses with no significant cytotoxicity. Molecular docking reveals no major change of the binding mode relative to that of their parent compounds, further supporting our design strategy. **Conclusions:** These compounds are structurally novel p97 inhibitors that display low toxicity and possess promising antiviral activity against SARS-CoV-2 and potentially other viruses. Further structural exploration is therefore justified and improved analogs will serve as useful tools for studying p97 as a promising host antiviral target.

## 1. Introduction

p97, also known as valosin-containing protein (VCP), belongs to the AAA+ (ATPase associated with various cellular activities) ATPase family [1]. p97 is a homohexamer and each subunit contains an N-domain, two ATPase domains (D1 and D2), and a short unstructured C-terminal tail [2]. The N-domain is flexible and can therefore engage in interactions with varied adaptor proteins, leading to subcellular localization of p97 and the recognition of different substrates [3]. While the D1 domain is important for hexamer assembly and the overall structural integrity, the D2 domain provides the major mechanical force [2]. ATP binding to the D1 and D2 domains induces remarkable conformational changes of the N-domain and the overall hexamer. The D2 hydrolysis of ATP provides energy for the extraction of ubiquitinated proteins from membranes, cellular structures, or protein complexes, including endoplasmic reticulum-associated protein degradation (ERAD) [4], mitochondrial-associated degradation [5], and ribosome-associated degradation [6]. While p97 generally extracts, unfolds, and delivers proteins to the 26S proteasome for degradation, it participates in autophagy [7], another system of degradation and recycling. Furthermore, p97 is also involved in other cellular functions including DNA repair and cell cycle progression [8]. Since p97 is closely linked to protein quality control and homeostasis, the pharmaceutical inhibition of p97 has been actively pursued as an anticancer strategy, especially in cancers whose survival depends on cellular protein homeostasis [9]. Furthermore, since p97 gain-of-function mutants have been proposed as a cause of pathogenesis in inclusion body myopathy, Paget’s disease of bone, and frontotemporal dementia (IBMPFD) [10,11,12], the inhibition of p97 activity may be exploited as a therapeutic strategy for IBMPFD.

Studies have demonstrated that p97 is also involved in multiple stages of virus replication, including entry, genome production, and egress, as well as innate and adaptive immune responses [13]. Indeed, p97 is a pro-viral host factor for flaviviruses, including West Nile virus [14], yellow fever virus [15], Zika virus [16,17], Japanese encephalitis virus [18,19], and dengue virus [19,20]. Furthermore, p97 is important for the replication of hepatitis C virus (a member of *Flaviviridae*) [21,22], chikungunya virus and other alphaviruses [23], human cytomegalovirus [24], and respiratory syncytial virus [25]. More importantly, the pharmaceutical inhibition of p97 exerts antiviral effects against Zika virus [16], human cytomegalovirus [24,26], influenza A and B viruses [27], and respiratory syncytial virus [25]. Besides the aforementioned viruses, p97 is also important for the replication of coronaviruses. For instance, p97 is required for the exit from endosomes by infectious bronchitis virus (IBV) [28]. The advent of the pandemic of coronavirus disease 2019 (COVID-19) has strengthened the interest in studying the roles of p97 in coronavirus replication and potential therapeutic applications of p97 inhibitors. p97 is linked to host cell proteostasis during SARS-CoV-2 replication [29]. It also impacts virus uncoating and viral RNA replication in human coronaviruses [30]. Remarkably, the p97 inhibitor NMS-873 exhibits excellent antiviral activity against SARS-CoV-2 [29], the causative agent of the COVID-19. In addition, NMS-873 and CB-5083 also inhibit human coronaviruses [30,31]. Interestingly, another inhibitor UPCDC-30245 blocks endo-lysosomal degradation and subsequent human coronavirus entry most likely through its basic sidechain [31]. Taken together, p97 is a promising host antiviral target, which can potentially be exploited to discover broad-spectrum antiviral agents against important viral pathogens including SARS-CoV-2.

**Figure 1:**
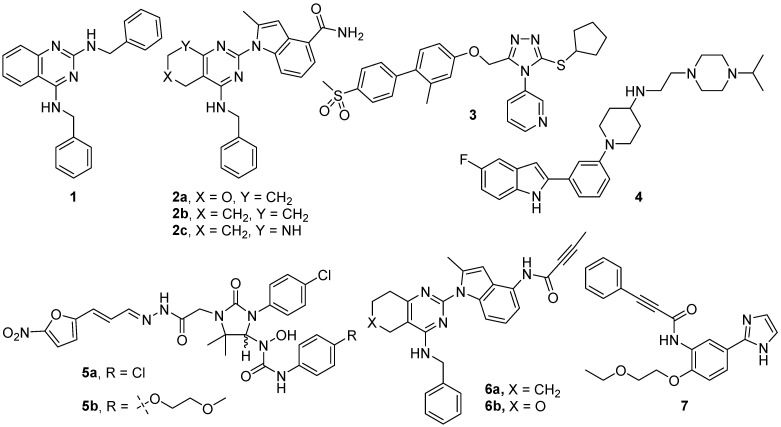
Selected p97 inhibitors.

p97’s implication in different human diseases and its therapeutic potential have spurred a growing interest in the discovery and development of p97 inhibitors, which have been recently reviewed [32,33,34]. p97 inhibitors can be categorized into three classes, namely ATP-competitive, allosteric, and covalent [34]. Some representative p97 inhibitors are shown in Figure 1. Among the ATP-competitive inhibitors, the prototypic inhibitor *N*^2^,*N*^4^-Dibenzylquinazoline-2,4-diamine (**1**, DBeQ) contains a quinazoline scaffold and binds to both the D2 and D1 domains [35]. Subsequent structure–activity relationship (SAR) studies lead to ML240 and ML241, which selectively target the D2 domain [36]. Further efforts have culminated in the discovery of the first clinical candidate CB-5083 (**2a**). This compound and its analog **2b** exemplify a new class of p97 inhibitors built on a pyrimidine core structure [37,38]. Further modification of the central bicyclic ring gives rise to another clinical candidate CB-5339 (**2c**) that lacks the side effects of CB-5083 [39]. Additional CB-5083-like inhibitors [40,41,42] and compounds based on a benzylquinazoline core [43,44] as well as a tri-substituted pyrimidine ring [45] have also been reported. NMS-873 (**3**) features an alkylsulfanyl-1,2,4-triazole core. As a prominent example of allosteric inhibitors, it binds into a tunnel between the D1 and D2 domains [46,47]. Optimization of 1,2,4-triazole-based p97 inhibitors leads to UPCDC-30766 with nanomolar biochemical and cell-based potency [48]. UPCDC30245 (**4**) is another allosteric inhibitor targeting a different site at the interface of the D1 and D2 domains [49]. Eeyarestatin I (**5a**, EerI) is the first reported p97 inhibitor [50], with its nitrofuran-containing group and aromatic moiety interacting with p97 and the hydrophobic membranes, respectively [51]. We have reported the derivative **5b** with improved aqueous solubility [52]. While the exact mechanism of action remains to be elucidated, EerI is probably converted into an active species that exerts its anti-p97 effect [53]. An early covalent inhibitor NMS-859 contains an α-chloroacetamide warhead, which selectively modifies Cys522 within the D2 active site [46]. Covalent inhibitors based on β-nitrostyrene and pyrazolo[3,4-*d*]pyrimidine scaffolds have also been reported [54]. We have designed CB-5083-inspired covalent inhibitors **6a** (LC-1028) and **6b** by incorporating a but-2-ynamide warhead. Furthermore, we have revealed a novel binding mode that allows for a covalent modification of Cys522 within the D2 active site [55]. More recently, new covalent inhibitors targeting Cys522 have been reported. As exemplified by FL-18 (**7**) [56], these inhibitors feature a benpropargylamide warhead and subsequent SAR has been reported [57]. Here we report new p97 inhibitors by modifying the structures of compounds **6a** and **6b** as well as evaluation of their antiviral activity against SARS-CoV-2.

## 2. Results and Discussion

### 2.1. Inhibitor Design

**Figure 2:**
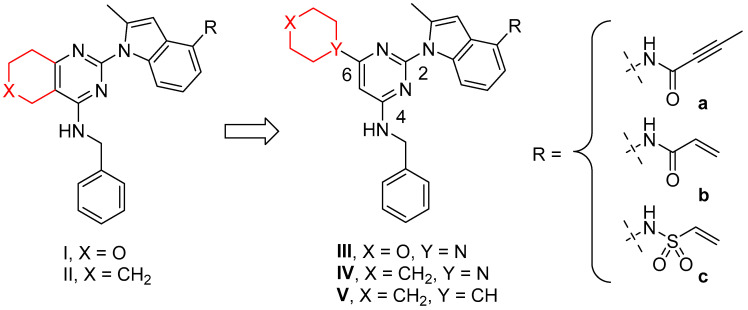
Design of new p97 inhibitors.

We aimed to test whether the central fused bicyclic ring in **I** and **II** could be rearranged and replaced by a bicyclic ring as shown in **III**–**V**, in which a morpholine, piperidine, or cyclohexyl ring was placed at position 6 to function as sidechains (Figure 2). A benzylamine and a functionalized indole were retained at position 4 and 2, respectively. Since we have demonstrated that but-2-ynamide (**a**) and acrylamide (**b**) are functional groups capable of engaging Cys522 in the p97 D2 active site, we decided to explore these two warheads. We also adopted vinyl sulfonamide (**c**) because it is a viable electrophilic warhead in the design of covalent inhibitors [58].

### 2.2. Biochemical Evaluation

The prepared new compounds **8a**–**10c** were first tested for the inhibition of p97 ATPase activity at 0.10 and 1.0 µM (Table 1). Known inhibitors **6a**–**b** as well as CB-5083 and NMS-873 were used as reference compounds. When tested at 0.10 µM, CB-5083 inhibited p97 by about 60% whereas NMS-873 caused poor inhibition. In comparison, compound **6a** exhibited inhibitory activity similar to that of CB-5083 while **6b** was weaker. All new analogs displayed excellent inhibition of p97 at 1.0 µM. However, when tested at 0.10 µM, compound **8a** maintained about 90% inhibition whereas **8b** and **8c** had notably reduced inhibitory activity. These results indicated that a but-2-ynamide functionality was preferred over acrylamide and vinyl sulfonamide. The same SAR trend was also observed in **9a**–**c** and **10a**–**c**. Remarkably, **9a** and **10a** inhibited p97 at >90% and close to 90%, respectively, even at 0.10 µM. Taken together, these biochemical results revealed that compounds **8a**, **9a**, and **10a** possessed improved inhibitory activity in comparison with their parent compounds **6a** and **6b**, supporting our design strategy as depicted in Figure 2. Furthermore, these compounds, especially **8a**, **9a**, and **10a**, exhibited enhanced inhibitory ability compared to known p97 inhibitors including CB-5083 and NMS-873. Therefore, they are new, potent p97 inhibitors.

Encouraged by this success, we attempted to apply the same strategy on CB-5083-like compounds, which contained a primary amide instead of an unsaturated amide warhead. Unfortunately, the resulting compounds **11** and **12** displayed minimal inhibition even at 1.0 µM (Table 1), a finding that was not unexpected. Our previous docking studies showed that compounds **6a** and **6b** adopted a binding mode different from that of CB-5083. As a result, the structural rearrangement was apparently not tolerated by the CB-5083-derived inhibitors.

### 2.3. Cytotoxicity

Because p97’s functions in cellular homeostasis are particularly critical for cancer development, p97 inhibitors have been mainly optimized as anticancer agents. In support of this notion, most p97 inhibitors show high cytotoxicity [34]. To see if the newly developed p97 inhibitors are also toxic to cancer cells, we first determined an inhibitor’s 50% cytotoxic concentration (CC_50_) value in A549/ACE2/TMPRSS2 cells [59]. Consistent with previous reports, CB-5083 was highly toxic whereas NMS-873 was approximately 4-fold less toxic. Known inhibitors **6a** and **6b** also showed significant cytotoxicity. Surprisingly, compounds **8a**–**c**, which contained a morpholine sidechain, were less toxic and had low µM CC_50_ values. Featuring a piperidine ring, compounds **9a**–**c** possessed further reduced cytotoxicity. Compounds **10a**–**c**, which had a cyclohexyl sidechain, showed similarly attenuated toxicity, with **10a** and **10b** having a CC_50_ value higher than 100 µM.

### 2.4. Anti-SARS-CoV-2 Activity

Since the newly developed p97 inhibitors are generally better tolerated by cells, we could test whether they have anti-SARS-CoV-2 activity (Table 1). We decided to use concentrations at 0.4 × CC_50_ for initial testing to avoid cytotoxicity caused by p97 inhibition. If a compound had a CC_50_ value higher than 100 µM, we would test it at 50 µM. Remdesivir was used as a control. A549/ACE2/TMPRSS2 cells were treated with a compound and inoculated at a multiplicity of infection of 0.01 for two days. The cultures were fixed and indirect immunofluorescence was performed using an antibody to the SARS-CoV-2 N protein. The percent infected cells were counted in compound-treated and vehicle-treated (DMSO) wells. SARS-CoV-2 inhibitors, such as Remdesivir, will reduce infectivity and spread of infection resulting in fewer N-protein-expressing cells. Known p97 inhibitors including CB-5083, NMS-873, as well as **6a** and **6b** exhibited minimal inhibition. The lack of antiviral activity by NMS-873 was in contrast to its excellent potency reported previously [29]. This discrepancy was likely due to the different cell lines used in the antiviral assays. Compounds **8a**–**c** also lacked antiviral activity. In contrast, compounds **9a** and **10a** displayed excellent antiviral activity at 0.4 × CC_50_ concentrations. Therefore, we proceeded to determine their EC_50_ values. Compound **9a** had an EC_50_ value of 6.7 µM, which was almost identical to that of **10a**. In comparison, **9b** and **10b** possessed markedly reduced, albeit similar, antiviral activity. Furthermore, **9c** and **10c** showed marginal inhibition. Taken together, evaluation of cytotoxicity and antiviral activity led to new inhibitors **9a** and **10a** that possessed lower toxicity and good antiviral activity against SARS-CoV-2.

### 2.5. Summary of SAR

**Figure 3:**
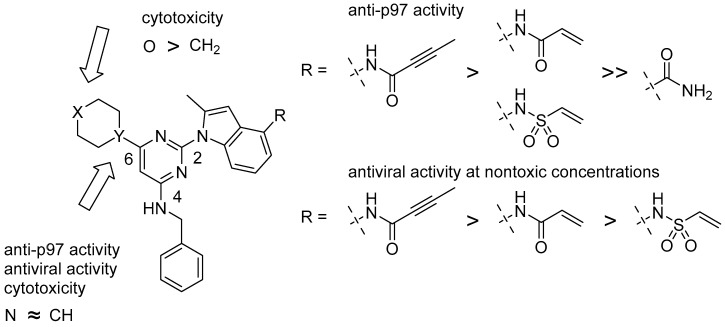
SAR summary.

In short, our SAR studies showed that but-2-ynamide was preferred over acrylamide and vinyl sulfonamide for anti-p97 activity whereas a primary amide ablated the inhibitory activity (Figure 3). To obtain good antiviral activity against SARS-CoV-2 at nontoxic concentrations, but-2-ynamide was also desirable. Brief exploration of the sidechain revealed that a terminal oxygen atom was detrimental, and a nonpolar methylene terminus was highly desired for reduced cytotoxicity. On the other hand, a proximal nitrogen atom on the sidechain had limited effects on the anti-p97 activity, antiviral activity, or cytotoxicity.

Examination of the structural features of **9a** and **10a** vs. **8a** revealed that the first two inhibitors shared a nonpolar methylene terminus as evidenced in the piperidine and cyclohexyl sidechains. However, the mechanism through which such minor structural variation elicited differential antiviral and cytotoxic effects was unknown. p97 is associated with a wide range of cellular functions and involved in multiple stages of viral replication. It is possible that a combination of a relatively less polar sidechain and a but-2-ynamide functionality allows compound **9a** and **10a** to selectively engage a set of adapter proteins. As a result, these compounds might disrupt p97-associated cellular functions that are key for viral replication without affecting other crucial functions. However, selective inhibition of p97-associated cellular functions by our compounds is speculative and the exact mechanism needs to be elucidated.

### 2.6. Molecular Docking

**Figure 4:**
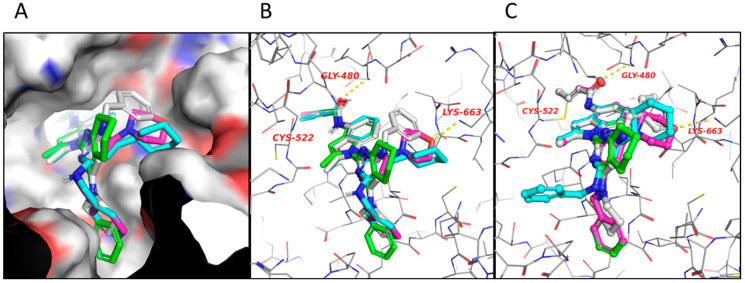
Potential binding modes and interactions of selected p97 inhibitors in human p97 (PDB 5FTK). (**A**) Binding modes of compounds **6a** (green), **8a** (magenta), **9a** (gray), and **10a** (cyan) at the active site of the D2 domain (surface). (**B**) Binding modes of compounds **6a** (green), **8a** (magenta), **9a** (gray), and **10a** (cyan) at the active site of the D2 domain. (**C**) Binding modes of compounds **6a** (green), **8a** (magenta), **9a** (gray), and **10a** (cyan) at the active site of the D2 domain after formation of a covalent bond with Cys522.

To obtain insight into the binding mode of compounds **8a**, **9a**, and **10a**, we performed molecular docking using a cryo-EM structure of hexameric human p97 (PDB 5FTK) that contains subunits A–F [49]. We have previously demonstrated that a neighboring subunit influences an inhibitor’s binding mode [55]. Therefore, we used a docking grid encompassing the D2 active site of subunit E (occupied by ADP in 5FTK) and the surrounding residues including those from subunit D. As previously reported, compound **6a** (green, Figure 4A,B) displayed a unique binding mode. Its indole moiety was projected into the narrow cavity that is normally targeted by the adenine ring of ADP. The amide functionality snugly fitted into a small tunnel at the back of the adenine-binding cavity. The amide carbonyl participated in a hydrogen bond with the backbone NH of Gly480 and the alkyne group was involved in lipophilic interactions with the surrounding residues. This binding orientation positioned the acetylene functionality close to Cys522. At the same time, the phenyl ring (of the benzylamine functionality) was positioned between adjacent subunits D and E, engaging in interactions with the neighboring residues including Pro636 from the subunit D. Inhibitor **8a** (magenta) exhibited a binding mode similar to that of **6a**. It formed a hydrogen bond with Gly480, and its acetylene group was equally close to Cys522. The phenyl ring was also placed between subunits D and E. On the other hand, the morpholine ring was projected away from the indole ring and the terminal oxygen atom formed a hydrogen bond with Lys663. Interestingly, docking of inhibitor **9a** (gray) placed its piperidine ring between subunits D and E, a position that was occupied by the phenyl ring in compounds **6a** and **8a**. Since the piperidine ring and the benzylamine functionality did not differ drastically in volume, inhibitor **9a** appeared to display an overall configuration similar to that of compound **8a**. We also studied inhibitor **10a** (cyan), which adopted the same binding mode as **8a**, with the phenyl being sandwiched between subunits D and E. Taken together, our docking study supported our design strategy that was based on a rearrangement of the central fused ring seen in compounds **6a** and **6b**. We also observed that p97 was accommodating when targeted by these p97 inhibitors. This flexibility is also supported by the fact that Cys522 could be modified by structurally diverse inhibitors [46,55,56] and can be exploited to design new analogs.

We have previously shown that the close proximity between Cys522 and the acetylene functionality in compound **6a** allows for a nucleophilic attack by Cys522 and subsequent formation of a covalent bond [55]. To examine such possibility for inhibitors **8a**, **9a**, and **10a**, we performed a covalent docking [60] using Cys522 as a nucleophile. Our study showed that a covalent bond formation was indeed feasible for all these three inhibitors (Figure 4C). Comparison of the binding modes before and after covalent bond formation revealed no major change of compound orientation or disruption of hydrogen bonding, with the exception that the phenyl ring in inhibitor **10a** flipped and was now mainly exposed to solvent.

### 2.7. Synthesis

**Scheme 1:**
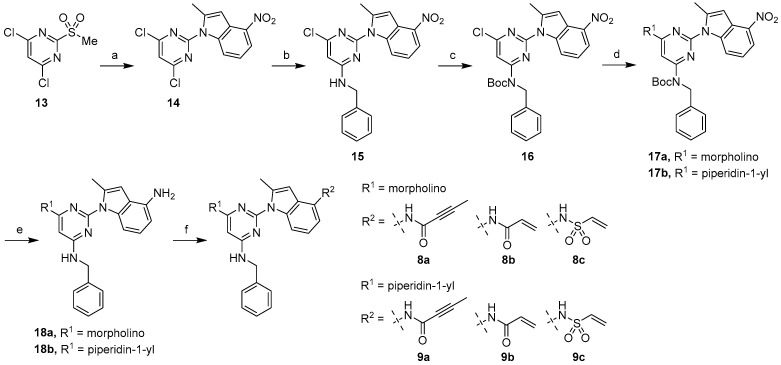
Synthesis of compounds **8a**–**c** and **9a**–**c**: (a) 2-methyl-4-nitro-1*H*-indole, NaH, DMF/THF, –60 °C to 0 °C; (b) BnNH_2_, DIPEA, dioxane, 100 °C; (c) Boc_2_O, DMAP, CH_3_CN; (d) morpholine or piperidine, dioxane, 100 °C; (e) SnCl_2_, EtOH, 70 °C; (f) but-2-ynoic acid, EDC, HOBt, DMF; or acryloyl chloride, K_2_CO_3_, acetone/H_2_O, 0 °C; or 2-chloroethane-1-sulfonyl chloride, Et_3_N, CH_2_Cl_2_.

**Scheme 2:**
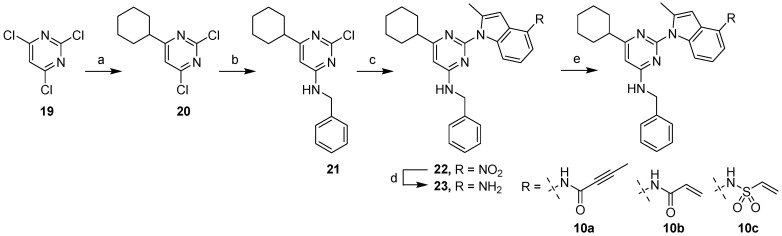
Synthesis of compounds **10a**–**c**: (a) cyclohexylzinc bromide, Pd(PPh_3_)_4_, 70 °C; (b) BnNH_2_, Et_3_N, MeOH; (c) 2-methyl-4-nitro-1*H*-indole, Pd_2_(dba)_3_, XPhos, Cs_2_CO_3_, dioxane, 100 °C; (d) SnCl_2_, MeOH, 75 °C; (e) but-2-ynoic acid, EDC, HOBt, DMF; or acryloyl chloride, K_2_CO_3_, acetone/H_2_O, 0 °C; or 2-chloroethane-1-sulfonyl chloride, Et_3_N, CH_2_Cl_2_.

The synthesis of compounds **8a**–**c** and **9a**–**c** started with commercially available 4,6-dichloro-2-(methylsulfonyl)pyrimidine (**13**, Scheme 1), whose sulfone has been reported to be selectively displaced by anionic nucleophiles [61]. Accordingly, intermediate **14** was obtained in excellent yield without column chromatography. Displacing one of the two chloro groups afforded benzylamine **15**. To attenuate the basicity of this molecule and set the stage for another nucleophilic displacement, the benzylamine functionality was protected with Boc to give chloride **16**. Subsequent displacement by morpholine gave rise to tri-substituted pyrimidine **17a** in a good yield. A reduction in the nitro group and concomitant removal of Boc under acidic reaction conditions led to aniline **18a**, which was further derivatized to give compounds **8a**–**c**. Analogously, chloride **16** was treated with piperidine to afford nitro **17b**. Reduction and incorporation of warheads led to compounds **9a**–**c** in modest to good yields. The synthesis of compounds **10a**–**c** began with 2,4,6-trichloropyrimidine (**19**, Scheme 2), to which a cyclohexyl group was incorporated via a Negishi coupling following procedures from the literature [62]. The resulting dichloro **20** was treated with benzylamine to give intermediate **21** in modest yield. A palladium-mediated C-N coupling was used to introduce the requisite nitroindole ring in **22**. A reduction in the nitro group gave aniline **23**, which was then converted into compounds **10a**–**c** in varied yields.

**Scheme 3:**
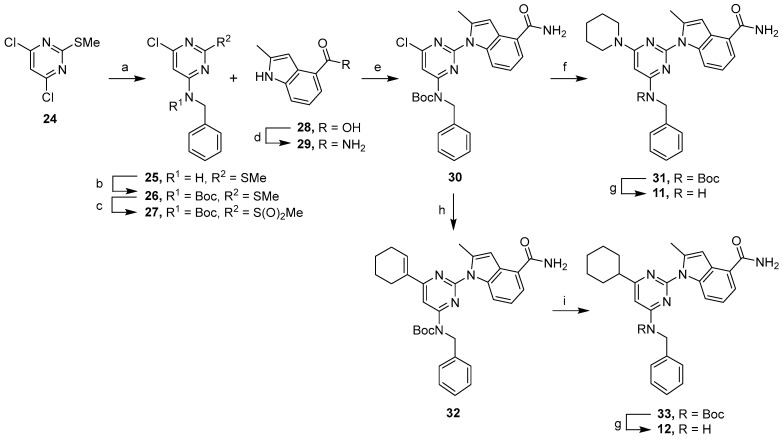
Synthesis of compounds **11** and **12**: (a) BnNH_2_, Et_3_N, THF, 70 °C; (b) Boc_2_O, DMAP, THF; (c) mCPBA, CH_2_Cl_2_; (d) Boc_2_O, Et_3_N, DMAP, THF; then 7 N NH_3_/MeOH; (e) LDA, THF; (f) piperidine, dioxane, 100 °C; (g) TFA, CH_2_Cl_2_; (h) cyclohex-1-en-1-ylboronic acid, Pd(PPh_3_)_2_Cl_2_, Na_2_CO_3_, dioxane/H_2_O, 100 °C; (i) Pd/C, H_2_, MeOH.

The synthesis of compounds **11** and **12** started with 4,6-dichloro-2-(methylthio)pyrimidine (**24**, Scheme 3), which was converted into intermediate **25** following procedures from the literature [45]. Protection of the benzylamine functionality with Boc and oxidation of the sulfide afforded sulfone **27** in excellent yield over two steps. The activated sulfone was subsequently displaced with deprotonated indole amide **29**, which was prepared from known indole acid **28** [63]. The resulting chloride **30** was treated with piperidine followed by deprotection of the Boc group to give compound **11** in a modest yield over two steps. To obtain compound **12**, a cyclohex-1-ene group was appended via a Suzuki coupling to give intermediate **32**. Hydrogenation of the cyclohexene ring and subsequent deprotection afforded compound **12** in very good yield over two steps.

## 3. Materials and Methods

### 3.1. Chemical Synthesis

General Methods: All commercial reagents were used as provided unless otherwise indicated. An anhydrous solvent dispensing system (J. C. Meyer) using 2 packed columns of neutral alumina was used for drying THF, Et_2_O, and CH_2_Cl_2,_ whereas 2 packed columns of molecular sieves were used to dry DMF. Solvents were dispensed under argon. Flash chromatography was performed with RediSep R_f_ silica gel columns on a Teledyne ISCO CombiFlash^®^ R_f_ system (Teledyne Labs, Thousand Oaks, CA, USA) using the solvents as indicated. Nuclear magnetic resonance spectra were recorded on a Varian 600 MHz (Varian, Inc., Palo Alto, CA, USA) or Bruker 400 MHz spectrometer (Bruker, Billerica, MA, USA) with Me_4_Si or signals from residual solvent as the internal standard for ^1^H or ^13^C. Chemical shifts are reported in ppm, and signals are described as s (singlet), d (doublet), t (triplet), q (quartet), m (multiplet), br s (broad singlet), and dd (double doublet). Values given for coupling constants are first order. High resolution mass spectra were recorded on an Agilent TOF II TOF/MS instrument equipped with either an ESI or APCI interface at the Center for Drug Design, University of Minnesota (Minneapolis, MN, USA).

#### 3.1.1. 1-(4,6-Dichloropyrimidin-2-yl)-2-methyl-4-nitro-1H-indole (**14**)

To a solution of 2-methyl-4-nitro-1*H*-indole (352 mg, 2.00 mmol) in anhydrous DMF (2 mL) and THF (3 mL) at rt was added NaH (160 mg, 60% dispersion, 4.00 mmol) in portions. After being stirred at rt for 5 min, the mixture was cooled at –60 °C and a solution of **13** (685 mg, 3.02 mmol) in anhydrous THF (4 mL) was added dropwise. The reaction mixture was allowed to warm to 0 °C and stir at 0 °C for 30 min. After being quenched with acetic acid (0.5 mL) and H_2_O (10 mL), the organic solvent was removed and additional H_2_O was added. The solid formed was filtered, washed with H_2_O, and dried to give compound **14** as a yellow solid (650 mg, 100%). ^1^H NMR (400 MHz, CDCl_3_) δ 8.72 (d, *J* = 8.3 Hz, 1H), 8.16 (d, *J* = 8.2 Hz, 1H), 7.32 (t, *J* = 8.2 Hz, 1H), 7.26–7.25 (m, 2H), 2.81 (s, 3H). ^13^C NMR (100 MHz, CDCl_3_) δ 162.4, 156.8, 143.2, 139.6, 138.5, 124.7, 122.5, 121.4, 119.9, 117.1, 108.3, 17.7. HRMS (APCI^+^) calcd for C_13_H_9_Cl_2_N_4_O_2_ (M + H)^+^ 323.0097, found 323.0106.

#### 3.1.2. N-Benzyl-6-chloro-2-(2-methyl-4-nitro-1H-indol-1-yl)pyrimidin-4-amine (**15**)

To a suspension of **14** (640 mg, 1.98 mmol) in anhydrous dioxane (10 mL) were added benzylamine (0.43 mL, 3.94 mmol) and DIPEA (0.69 mL, 3.96 mmol). The mixture was heated at 80 °C for 18 h, allowed to cool to rt, and poured into H_2_O (100 mL). The resulting mixture was stirred for 40 min and the organic solvent was removed in vacuo. The solid formed was filtered, washed with H_2_O, and dried to give compound **15** as a yellow solid (795 mg, 100%). ^1^H NMR (400 MHz, DMSO-*d*_6_) δ 8.67 (t, *J* = 5.8 Hz, 1H), 8.25 (d, *J* = 8.1 Hz, 1H), 8.04 (d, *J* = 8.0 Hz, 1H), 7.37–7.33 (m, 4H), 7.30–7.28 (m, 1H), 7.19–7.15 (m, 1H), 7.03 (s, 1H), 6.63 (s, 1H), 4.62 (d, *J* = 5.7 Hz, 2H), 2.60 (s, 3H). ^13^C NMR (100 MHz, DMSO-*d*_6_) δ 164.0, 156.8, 155.9, 143.2, 138.6, 138.3, 137.9, 128.5, 127.1, 127.0, 122.7, 121.3, 120.9, 118.5, 104.8, 101.3, 44.0, 16.1. HRMS: no desired ionization observed in ESI or APCI mode.

#### 3.1.3. tert-Butyl Benzyl(6-chloro-2-(2-methyl-4-nitro-1H-indol-1-yl)pyrimidin-4-yl)carbamate (**16**)

To a suspension of **15** (790 mg, 2.00 mmol) in anhydrous CH_3_CN (20 mL) were added Boc_2_O (506 mg, 2.32 mmol) and DMAP (14 mg, 0.11 mmol). The red mixture was allowed to stir at rt for 2 h and the reaction was quenched with H_2_O (20 mL). After the organic solvent was removed in vacuo, the mixture was extracted with CH_2_Cl_2_ (40 mL). The combined organic layer was washed with H_2_O (60 mL × 2), dried over Na_2_SO_4_, and filtered. The filtrate was concentrated, and the residue was purified by flash column chromatography (5–50% EtOAc/hexanes) to give compound **16** as a yellow solid (670 mg, 68%). ^1^H NMR (400 MHz, CDCl_3_) δ 8.17–8.16 (m, 2H), 8.01 (d, *J* = 8.1 Hz, 1H), 7.34 (t, *J* = 7.3 Hz, 2H), 7.30–7.26 (m, 1H), 7.19 (d, *J* = 7.6 Hz, 2H), 7.08 (s, 1H), 6.95 (t, *J* = 8.2 Hz, 1H), 5.31 (s, 2H), 2.55 (s, 3H), 1.46 (s, 9H). ^13^C NMR (100 MHz, CDCl_3_) δ 162.3, 161.6, 155.7, 153.0, 142.7, 139.1, 138.4, 137.6, 128.7, 127.2, 125.8, 123.9, 121.5, 120.6, 118.9, 108.4, 106.6, 84.1, 49.1, 27.9, 16.8. HRMS: no desired ionization observed in ESI or APCI mode.

#### 3.1.4. tert-Butyl Benzyl(2-(2-methyl-4-nitro-1H-indol-1-yl)-6-morpholinopyrimidin-4-yl)carbamate (**17a**)

A solution of **16** (195 mg, 0.395 mmol) and morpholine (0.11 mL, 1.26 mmol) in anhydrous dioxane (4 mL) was heated at 100 °C for 4 h. The mixture was allowed to cool to rt, and H_2_O (6 mL) was added. The resulting mixture was stirred for 1 h and the organic solvent was removed in vacuo. The solid formed was filtered, washed with H_2_O, and dried to give compound **17a** as a yellow solid (180 mg, 84%). ^1^H NMR (400 MHz, CDCl_3_) δ 8.03 (dd, *J* = 8.2, 3.5 Hz, 2H), 7.39 (s, 1H), 7.33–7.29 (m, 2H), 7.26–7.24 (m, 1H), 7.21–7.19 (m, 2H), 7.09 (s, 1H), 6.93 (t, *J* = 8.2 Hz, 1H), 5.28 (s, 1H), 3.82 (t, *J* = 4.8 Hz, 4H), 3.70 (t, *J* = 4.8 Hz, 4H), 2.57 (s, 3H), 1.42 (s, 9H). ^13^C NMR (100 MHz, CDCl_3_) δ 164.5, 161.2, 155.5, 153.9, 142.8, 139.1, 139.0, 138.8, 128.5, 126.9, 126.1, 123.6, 120.6, 120.2, 118.4, 105.1, 90.1, 82.7, 66.6, 48.8, 44.9, 28.1, 16.5. HRMS: no desired ionization observed in ESI or APCI mode.

#### 3.1.5. 1-(4-(Benzylamino)-6-morpholinopyrimidin-2-yl)-2-methyl-1H-indol-4-amine (**18a**)

To a suspension of **17a** (170 mg, 0.312 mmol) in EtOH (6mL), SnCl_2_ (412 mg, 2.17 mmol) was added, and the mixture was heated at 70 °C for 35 h. After being allowed to cool to rt, the mixture was diluted with EtOAc (20 mL), quenched with saturated NaHCO_3_ (12 mL) plus H_2_O (3 mL), and filtered through a pad of Celite. The organic layer of the filtrate was separated, washed with brine (20 mL), dried over Na_2_SO_4_, and filtered. The filtrate was concentrated, and the residue was purified by flash column chromatography (0–5% MeOH/CH_2_Cl_2_) to give compound **18a** as a pale solid (57 mg, 44%). ^1^H NMR (400 MHz, CD_3_OD) δ 7.35–7.29 (m, 5H), 7.25–7.20 (m, 1H), 6.80 (t, *J* = 7.9 Hz, 1H), 6.42 (dd, *J* = 7.5, 0.8 Hz, 1H), 6.36–6.35 (m, 1H), 5.52 (s, 1H), 4.55 (s, 2H), 3.72 (t, *J* = 4.6 Hz, 4H), 3.50 (t, *J* = 5.2 Hz, 4H), 2.52 (s, 3H). ^13^C NMR (100 MHz, CD_3_OD) δ 166.1, 165.2, 158.3, 141.1, 139.6, 139.2, 136.4, 129.5, 128.1, 127.9, 123.6, 119.7, 107.6, 106.2, 102.3, 80.0, 67.6, 46.0, 45.8, 16.3. HRMS (ESI^+^) calcd for C_24_H_27_N_6_O (M + H)^+^ 415.2241, found 415.2246.

#### 3.1.6. N-(1-(4-(Benzylamino)-6-morpholinopyrimidin-2-yl)-2-methyl-1H-indol-4-yl)but-2-ynamide (**8a**)

To a solution of **18a** (48 mg, 0.12 mmol) in anhydrous DMF (3 mL), but-2-ynoic acid (15 mg, 0.18 mmol), EDC (45 mg, 0.24 mmol), and HOBt (16 mg, 0.12 mmol) were added. The mixture was stirred at rt for 70 h and H_2_O (18 mL) was added while stirring. The solid formed was filtered, washed with H_2_O, and dried. The residue was purified by flash column chromatography (0–5% MeOH/CH_2_Cl_2_) to give compound **8a** as a yellowish solid (35 mg, 63%). ^1^H NMR (400 MHz, CDCl_3_) δ 7.85 (d, *J* = 8.4 Hz, 1H), 7.72 (d, *J* = 7.9 Hz, 1H), 7.56 (s, 1H), 7.38–7.35 (m, 2H), 7.34–7.33 (m, 2H), 7.32–7.29 (m, 1H), 7.07 (t, *J* = 8.1 Hz, 1H), 6.28 (s, 1H), 5.33 (s, 2H), 4.51 (d, *J* = 5.9 Hz, 2H), 3.76 (t, *J* = 4.8 Hz, 4H), 3.54 (t, *J* = 4.9 Hz, 4H), 2.63 (s, 3H), 2.01 (s, 3H). ^13^C NMR (100 MHz, CDCl_3_) δ 164.4, 164.2, 156.8, 151.1, 138.5, 137.7, 137.6, 128.9, 127.9, 127.6, 127.3, 122.2, 120.7, 116.5, 113.4, 111.4, 100.6, 84.2, 75.8, 66.6, 45.8, 44.8, 16.5, 3.9. HRMS (ESI^+^) calcd for C_28_H_29_N_6_O_2_ (M + H)^+^ 481.2347, found 481.2341.

#### 3.1.7. N-(1-(4-(Benzylamino)-6-morpholinopyrimidin-2-yl)-2-methyl-1H-indol-4-yl)acrylamide (**8b**)

To a solution of K_2_CO_3_ (46 mg, 0.33 mmol) in H_2_O (0.5 mL) and acetone (1 mL) at 0 °C, acryloyl chloride (27 µL, 0.33 mmol) was added and then dropwise **18a** (62 mg, 0.15 mmol) in acetone (1.5 mL) was added. After the mixture was allowed to stir at 0 °C for 1 h and subsequently at rt for 1 h, H_2_O (6 mL) was added. The resulting mixture was extracted with 2% MeOH/CH_2_Cl_2_ (10 mL × 5) and the combined organic layer was washed with brine (30 mL), dried over Na_2_SO_4_, and filtered. The filtrate was concentrated, and the residue was purified by flash column chromatography (0–5% MeOH/CH_2_Cl_2_) to give compound **8b** as a pale solid (51 mg, 72%). ^1^H NMR (400 MHz, CDCl_3_) δ 7.87 (d, *J* = 8.4 Hz, 1H), 7.83 (brs, 1H), 7.38–7.27 (m, 6H), 7.09 (t, *J* = 8.0 Hz, 1H), 6.44 (dd, *J* = 16.9, 1.6 Hz, 1H), 6.35 (d, *J* = 10.3 Hz, 1H), 6.28 (s, 1H), 5.75 (d, *J* = 10.0 Hz, 1H), 5.33 (s, 1H), 4.51 (s, 2H), 3.77 (t, *J* = 4.5 Hz, 4H), 3.54 (t, *J* = 4.9 Hz, 4H), 2.63 (s, 3H). ^13^C NMR (100 MHz, CDCl_3_) δ 164.4, 164.2, 163.4, 156.8, 138.5, 137.6, 131.5, 128.9, 128.6, 128.3, 127.6, 127.34, 127.33, 122.3, 121.0, 113.2, 111.2, 100.6, 78.3, 66.7, 45.7, 44.8, 16.5. HRMS (ESI^+^) calcd for C_27_H_29_N_6_O_2_ (M + H)^+^ 469.2347, found 469.2342.

#### 3.1.8. N-(1-(4-(Benzylamino)-6-morpholinopyrimidin-2-yl)-2-methyl-1H-indol-4-yl)ethenesulfonamide (**8c**)

To a solution of **18a** (62 mg, 0.15 mmol) in anhydrous CH_2_Cl_2_ (3 mL), Et_3_N (17 µL, 0.16 mmol) and 2-chloroethane-1-sulfonyl chloride (42 µL, 0.30 mmol) were added. After the mixture was allowed to stir at rt for 1h, additional Et_3_N (17 µL, 0.16 mmol) was added and the mixture was stirred at rt for 4 h. H_2_O (5 mL) was added to quench the reaction, and the resulting mixture was extracted with CH_2_Cl_2_ (10 mL × 3). The combined organic layer was concentrated, and the residue was purified by flash column chromatography (10–90% EtOAc/hexanes) to give compound **8c** as a pale solid (57 mg, 75%). ^1^H NMR (400 MHz, CD_3_OD) δ 7.68 (d, *J* = 8.3 Hz, 1H), 7.34–7.29 (m, 4H), 7.25–7.21 (m, 1H), 7.02 (dd, *J* = 7.7, 0.9 Hz, 1H), 6.92 (t, *J* = 8.0 Hz, 1H), 6.64 (dd, *J* = 16.5, 10.0 Hz, 1H), 6.47 (t, *J* = 1.0 Hz, 1H), 6.02 (d, *J* = 16.5 Hz, 1H), 5.81 (d, *J* = 10.0 Hz, 1H), 5.57 (s, 1H), 4.55 (s, 2H), 3.72 (t, *J* = 4.9 Hz, 4H), 3.50 (t, *J* = 4.9 Hz, 4H), 2.53 (s, 3H). ^13^C NMR (100 MHz, CD_3_OD) δ 166.1, 165.2, 157.9, 139.1, 138.8, 137.5, 129.7, 129.5, 128.5, 128.2, 128.1, 128.0, 127.1, 125.6, 122.7, 117.3, 112.9, 102.8, 67.6, 46.0, 45.7, 16.3. HRMS (ESI^+^) calcd for C_26_H_29_N_6_O_3_S (M + H)^+^ 505.2016, found 505.2015.

#### 3.1.9. tert-Butyl Benzyl(2-(2-methyl-4-nitro-1H-indol-1-yl)-6-(piperidin-1-yl)pyrimidin-4-yl)carbamate (**17b**)

In a manner similar to that described for compound **17a**, **16** (346 mg, 0.700 mmol) was treated with piperidine (0.21 mL, 2.12 mmol) to give compound **17b** as a yellow solid (371 mg, 98%). ^1^H NMR (400 MHz, CDCl_3_) δ 8.09 (d, *J* = 8.2 Hz, 1H), 8.04 (d, *J* = 8.0 Hz, 1H), 7.33 (s, 1H), 7.30 (d, *J* = 7.6 Hz, 2H), 7.26–7.22 (m, 3H), 7.10 (s, 1H), 6.94 (t, *J* = 8.2 Hz, 1H), 5.27 (s, 2H), 3.70 (t, *J* = 5.2 Hz, 4H), 2.60 (s, 3H), 1.74–1.64 (m, 6H), 1.44 (s, 9H). ^13^C NMR (100 MHz, CDCl_3_) δ 163.8, 161.0, 155.6, 153.8, 143.0, 139.1, 139.0, 138.8, 128.4, 126.7, 126.2, 123.6, 120.44, 120.35, 118.2, 104.8, 90.2, 82.4, 48.8, 45.8, 28.1, 25.7, 24.7, 16.4. HRMS: no desired ionization observed in ESI or APCI mode.

#### 3.1.10. 1-(4-(Benzylamino)-6-(piperidin-1-yl)pyrimidin-2-yl)-2-methyl-1H-indol-4-amine (**18b**)

In a manner similar to that described for compound **18a**, **17b** (353 mg, 0.650 mmol) was reduced to give compound **18b** as a pale solid (69 mg, 26%). ^1^H NMR (400 MHz, CD_3_OD) δ 7.32–7.27 (m, 5H), 7.23–7.18 (m, 1H), 6.80 (t, *J* = 7.9 Hz, 1H), 6.42 (d, *J* = 7.3 Hz, 1H), 6.34 (s, 1H), 5.46 (s, 1H), 4.49 (s, 2H), 3.50 (t, *J* = 5.4 Hz, 4H), 2.51 (s, 3H), 1.65–1.59 (m, 2H), 1.56–1.50 (m, 4H). ^13^C NMR (100 MHz, CD_3_OD) δ 166.0, 164.6, 158.2, 141.1, 139.5, 139.2, 136.4, 129.5, 128.1, 127.9, 123.5, 119.6, 107.5, 106.2, 102.0, 79.5, 46.7, 45.8, 26.5, 25.8, 16.2. HRMS (APCI^+^) calcd for C_25_H_29_N_6_ (M + H)^+^ 413.2448, found 413.2443.

#### 3.1.11. N-(1-(4-(Benzylamino)-6-(piperidin-1-yl)pyrimidin-2-yl)-2-methyl-1H-indol-4-yl)but-2-ynamide (**9a**)

In a manner similar to that described for compound **8a**, **18b** (42 mg, 0.10 mmol) was couple with but-2-ynoic acid (13 mg, 0.16 mmol) to give compound **9a** as a yellowish solid (39 mg, 80%). ^1^H NMR (400 MHz, CDCl_3_) δ 7.87 (d, *J* = 8.3 Hz, 1H), 7.73 (d, *J* = 7.8 Hz, 1H), 7.54 (s, 1H), 7.38–7.32 (m, 4H), 7.31–7.28 (m, 1H), 7.07 (t, *J* = 8.1 Hz, 1H), 6.27 (s, 1H), 5.35 (s, 1H), 5.24 (s, 1H), 4.49 (d, *J* = 5.8 Hz, 2H), 3.56 (t, *J* = 5.4 Hz, 4H), 2.64 (s, 3H), 2.02 (s, 3H), 1.69–1.65 (m, 2H), 1.63–1.57 (m, 4H). ^13^C NMR (100 MHz, CDCl_3_) δ 164.3, 163.6, 156.8, 151.1, 138.7, 137.8, 137.6, 128.8, 127.9, 127.5, 127.4, 122.1, 120.6, 113.2, 111.4, 100.2, 84.1, 77.9, 75.9, 45.9, 45.7, 25.6, 24.8, 16.4, 3.9. HRMS (APCI^−^) calcd for C_29_H_29_N_6_O (M–H)^−^ 477.2408, found 477.2409.

#### 3.1.12. N-(1-(4-(Benzylamino)-6-(piperidin-1-yl)pyrimidin-2-yl)-2-methyl-1H-indol-4-yl)acrylamide (**9b**)

In a manner similar to that described for compound **8b**, **18b** (62 mg, 0.15 mmol) was treated with acryloyl chloride (27 µL, 0.33 mmol) to give compound **9b** as a pale solid (35 mg, 50%). ^1^H NMR (400 MHz, CD_3_OD) δ 7.69 (d, *J* = 8.3 Hz, 1H), 7.50 (d, *J* = 7.7 Hz, 1H), 7.34–7.29 (m, 4H), 7.24–7.21 (m, 1H), 6.97 (t, *J* = 8.1 Hz, 1H), 6.61 (dd, *J* = 16.9, 10.2 Hz, 1H), 6.44 (s, 1H), 6.38 (dd, *J* = 17.0, 1.8 Hz, 1H), 5.77 (dd, *J* = 10.2, 1.7 Hz, 1H), 5.53 (s, 1H), 4.52 (s, 2H), 3.53 (t, *J* = 5.4 Hz, 4H), 2.55 (s, 3H), 1.68–1.63 (m, 2H), 1.59–1.53 (m, 4H). ^13^C NMR (100 MHz, CD_3_OD) δ 166.4, 166.1, 164.6, 158.0, 141.2, 139.1, 138.4, 132.5, 129.53, 129.46, 128.1, 127.9, 127.6, 123.6, 122.5, 115.5, 112.3, 102.5, 79.7, 46.7, 45.8, 26.6, 25.9, 16.2. HRMS (APCI^−^) calcd for C_28_H_29_N_6_O (M–H)^−^ 465.2408, found 465.2407.

#### 3.1.13. N-(1-(4-(Benzylamino)-6-(piperidin-1-yl)pyrimidin-2-yl)-2-methyl-1H-indol-4-yl)ethenesulfonamide (**9c**)

In a manner similar to that described for compound **8c**, **18b** (62 mg, 0.15 mmol) was treated with 2-chloroethane-1-sulfonyl chloride (17 µL, 0.16 mmol) to give compound **9c** as a pale solid (48 mg, 64%). ^1^H NMR (400 MHz, CD_3_OD) δ 7.67 (d, *J* = 8.3 Hz, 1H), 7.31–7.30 (m, 2H), 7.29–7.26 (m, 2H), 7.24–7.18 (m, 1H), 7.02 (dd, *J* = 7.7, 0.9 Hz, 1H), 6.91 (t, *J* = 8.0 Hz, 1H), 6.62 (dd, *J* = 16.5, 10.0 Hz, 1H), 6.47–6.46 (m, 1H), 6.01 (d, *J* = 16.5 Hz, 1H), 5.77 (d, *J* = 10.0 Hz, 1H), 5.51 (s, 1H), 4.49 (s, 2H), 3.50 (t, *J* = 5.4 Hz, 4H), 2.53 (s, 3H), 1.65–1.60 (m, 2H), 1.56–1.50 (m, 4H). ^13^C NMR (100 MHz, CD_3_OD) δ 166.0, 164.5, 157.9, 141.1, 139.1, 138.8, 137.4, 129.7, 129.5, 128.4, 128.0, 127.9, 127.1, 125.6, 122.6, 117.2, 112.8, 102.6, 46.6, 45.8, 26.5, 25.8, 16.2. HRMS (APCI^−^) calcd for C_27_H_29_N_6_O_2_S (M–H)^−^ 501.2078, found 501.2079.

#### 3.1.14. N-Benzyl-2-chloro-6-cyclohexylpyrimidin-4-amine (**21**)

A solution of **20** [62] (1.36 g, 5.89 mmol), BnNH_2_ (0.79 mL, 7.23 mmol), and Et_3_N (1.25 mL, 8.96 mmol) in anhydrous MeOH (50 mL) was allowed to stir at rt for 2 h. After the organic solvent was removed in vacuo, H_2_O was added and the mixture was extracted with CH_2_Cl_2_. The organic layer was concentrated, and the residue was purified by flash column chromatography (25% EtOAc/hexanes) to give compound **21** as a white solid (870 mg, 49%). ^1^H NMR (400 MHz, CDCl_3_) δ 7.38–7.28 (m, 5H), 6.04 (s, 1H), 4.54–4.52 (2H), 2.47–2.40 (m, 1H), 1.89–1.86 (m, 2H), 1.82–1.78 (m, 2H), 1.73–1.68 (m, 1H), 1.42–1.30 (m, 4H), 1.28–1.16 (m, 1H). ^13^C NMR (100 MHz, CDCl_3_) δ 176.2, 164.4, 160.3, 137.4, 129.0, 127.9, 127.6, 98.2, 53.6, 45.8, 31.9, 26.3, 25.9. HRMS (ESI^+^) calcd for C_17_H_21_ClN_3_ (M + H)^+^ 302.1419, found 302.1418.

#### 3.1.15. N-Benzyl-6-cyclohexyl-2-(2-methyl-4-nitro-1H-indol-1-yl)pyrimidin-4-amine (**22**)

A mixture of **21** (400 mg, 1.32 mmol), 2-methyl-4-nitro-1*H*-indole (239 mg, 1.36 mmol), Pd_2_(dba)_3_ (125 mg, 0.136 mmol), XPhos (132 mg, 0.277 mmol), and Cs_2_CO_3_ (948 mg, 2.91 mmol) in anhydrous dioxane (13 mL) was degassed and heated at 100 °C for 2.5 h. After the organic solvent was removed in vacuo, H_2_O was added and the mixture was extracted with CH_2_Cl_2_. The organic layer was concentrated, and the residue was purified by flash column chromatography (25% EtOAc/hexanes) to give compound **22** as a yellow solid (410 mg, 70%). ^1^H NMR (400 MHz, CDCl_3_) δ 8.35 (d, *J* = 8.2 Hz, 1H), 8.10 (dd, *J* = 8.1, 0.9 Hz, 1H), 7.41–7.31 (m, 5H), 7.16–7.11 (m, 2H), 6.16 (s, 1H), 5.35 (brs, 1H), 4.64–4.62 (m, 2H), 2.72 (s, 3H), 2.59–2.52 (m, 1H), 2.00–1.96 (m, 2H), 1.90–1.85 (m, 2H), 1.79–1.73 (m, 1H), 1.57 (dd, *J* = 12.4, 3.1 Hz, 1H), 1.51 (dd, *J* = 12.5, 3.1 Hz, 1H), 1.43 (dt, *J* = 12.6, 3.3 Hz, 1H), 1.37 (dt, *J* = 12.5, 3.2 Hz, 1H), 1.33–1.26 (m, 1H). ^13^C NMR (100 MHz, CDCl_3_) δ 163.9, 156.9, 143.4, 139.2, 139.0, 129.0, 127.8, 127.4, 123.9, 120.7, 120.6, 119.9, 118.5, 117.6, 116.9, 105.1, 46.0, 45.6, 32.1, 26.4, 26.1, 16.7. HRMS (ESI^+^) calcd for C_26_H_28_N_5_O_2_ (M + H)^+^ 442.2238, found 442.2224.

#### 3.1.16. 1-(4-(Benzylamino)-6-cyclohexylpyrimidin-2-yl)-2-methyl-1H-indol-4-amine (**23**)

A suspension of **22** (398 mg, 0.902 mmol) and SnCl_2_ (1.21 g, 6.38 mmol) in MeOH (10 mL) was heated at 75 °C for 5 h. After the mixture was allowed to cool to rt, saturated NaHCO_3_ was added to quench the reaction. The resulting yellow slurry was filtered and washed with MeOH. The filtrate was extracted with CH_2_Cl_2_. The organic layer was concentrated, and the residue was purified by flash column chromatography (50% EtOAc/hexanes) to give compound **23** as a yellow solid (248 mg, 67%). ^1^H NMR (400 MHz, CDCl_3_) δ 7.63 (d, *J* = 8.3 Hz, 1H), 7.39–7.28 (m, 5H), 6.95 (t, *J* = 8.0 Hz, 1H), 6.44 (dd, *J* = 7.5, 0.8 Hz, 1H), 6.27 (s, 1H), 6.03 (s, 1H), 5.23 (brs, 1H), 4.60 (d, *J* = 5.7 Hz, 2H), 3.79 (s, 2H), 2.65 (s, 3H), 2.52 (tt, *J* = 11.6, 3.3 Hz, 1H), 2.00–1.94 (m, 2H), 1.88–1.83 (m, 2H), 1.77–1.73 (m, 1H), 1.58 (dd, *J* = 12.4, 3.2 Hz, 1H), 1.51 (dd, *J* = 12.5, 3.2 Hz, 1H), 1.42 (dt, *J* = 12.6, 3.2 Hz, 1H), 1.36 (dt, *J* = 12.3, 3.1 Hz, 1H), 1.32–1.24 (m, 1H). ^13^C NMR (100 MHz, CDCl_3_) δ 174.5, 163.8, 157.8, 138.5, 138.04, 137.97, 136.2, 128.9, 127.7, 127.6, 122.8, 118.2, 106.3, 105.8, 101.4, 97.1, 46.0, 45.6, 32.0, 26.5, 26.2, 16.7. HRMS (ESI^+^) calcd for C_26_H_30_N_5_ (M + H)^+^ 412.2496, found 412.2493.

#### 3.1.17. N-(1-(4-(Benzylamino)-6-cyclohexylpyrimidin-2-yl)-2-methyl-1H-indol-4-yl)but-2-ynamide (**10a**)

In a manner similar to that described for compound **8a**, **23** (37 mg, 0.090 mmol) was couple with but-2-ynoic acid (12 mg, 0.14 mmol) to give compound **10a** as a pale solid (13 mg, 30%). ^1^H NMR (400 MHz, CDCl_3_) δ 7.92 (d, *J* = 8.3 Hz, 1H), 7.74 (d, *J* = 7.8 Hz, 1H), 7.50 (s, 1H), 7.40–7.35 (m, 4H), 7.34–7.29 (m, 1H), 7.09 (t, *J* = 8.1 Hz, 1H), 6.31 (s, 1H), 6.09 (s, 1H), 5.24 (brs, 1H), 4.62 (d, *J* = 5.5 Hz, 2H), 2.67 (s, 3H), 2.53 (tt, *J* = 11.8, 3.5 Hz, 1H), 2.03 (s, 3H), 1.97 (d, *J* = 12.9 Hz, 3H), 1.87–1.83 (m, 2H), 1.80–1.73 (m, 1H), 1.57–1.49 (m, 2H), 1.44–1.33 (m, 2H), 1.31–1.25 (m, 1H). ^13^C NMR (100 MHz, CDCl_3_) δ 174.7, 163.8, 157.5, 151.1, 138.3, 138.1, 137.7, 129.0, 127.9, 127.8, 127.6, 122.4, 120.8, 113.5, 111.6, 100.8, 97.6, 84.2, 75.9, 46.0, 45.6, 32.1, 26.5, 26.2, 16.8, 4.0. HRMS: no desired ionization observed in ESI or APCI mode.

#### 3.1.18. N-(1-(4-(Benzylamino)-6-cyclohexylpyrimidin-2-yl)-2-methyl-1H-indol-4-yl)acrylamide (**10b**)

In a manner similar to that described for compound **8b**, **23** (27 mg, 0.066 mmol) was treated with acryloyl chloride (6.5 µL, 0.080 mmol) to give compound **10b** as a white solid (28 mg, 91%). ^1^H NMR (400 MHz, CDCl_3_) δ 7.92 (d, *J* = 8.3 Hz, 1H), 7.83 (d, *J* = 7.8 Hz, 1H), 7.44 (s, 1H), 7.38–7.27 (m, 5H), 7.09 (t, *J* = 8.2 Hz, 1H), 6.44 (dd, *J* = 16.9, 1.7 Hz, 1H), 6.35–6.29 (m, 2H), 6.07 (s, 1H), 5.75 (d, *J* = 10.0 Hz, 1H), 5.36 (brs, 1H), 4.58 (d, *J* = 5.6 Hz, 2H), 2.64 (s, 3H), 2.51 (tt, *J* = 11.7, 3.4 Hz, 1H), 1.98–1.93 (m, 2H), 1.87–1.82 (m, 2H), 1.77–1.71 (m, 1H), 1.55 (dd, *J* = 12.5, 3.2 Hz, 1H), 1.49 (dd, *J* = 12.5, 3.1 Hz, 1H), 1.41 (dt, *J* = 12.4, 3.2 Hz, 1H), 1.34 (dt, *J* = 12.3, 3.0 Hz, 1H), 1.30–1.23 (m, 1H). ^13^C NMR (100 MHz, CDCl_3_) δ 174.5, 163.8, 163.6, 157.4, 138.4, 137.9, 137.7, 131.6, 128.9, 128.3, 127.6, 127.5, 127.4, 122.4, 121.2, 113.5, 111.4, 100.9, 97.6, 46.0, 45.5, 32.0, 26.4, 26.1, 16.7. HRMS (APCI^−^) calcd for C_29_H_30_N_5_O (M–H)^−^ 464.2456, found 464.2454.

#### 3.1.19. N-(1-(4-(Benzylamino)-6-cyclohexylpyrimidin-2-yl)-2-methyl-1H-indol-4-yl)ethenesulfonamide (**10c**)

In a manner similar to that described for compound **8c**, **23** (62 mg, 0.15 mmol) was treated with 2-chloroethane-1-sulfonyl chloride (17 µL, 0.16 mmol) to give compound **10c** as a pale solid (43 mg, 57%). ^1^H NMR (400 MHz, CDCl_3_) δ 7.92 (d, *J* = 8.2 Hz, 1H), 7.40–7.29 (m, 5H), 7.11 (d, *J* = 7.6 Hz, 1H), 7.03 (t, *J* = 8.0 Hz, 1H), 6.79 (s, 1H), 6.53 (dd, *J* = 16.5, 9.9 Hz, 1H), 6.31 (s, 1H), 6.18 (d, *J* = 16.6 Hz, 1H), 6.10 (s, 1H), 5.80 (d, *J* = 9.9 Hz, 1H), 5.46 (brs, 1H), 4.60 (d, *J* = 5.7 Hz, 2H), 2.63 (s, 3H), 2.53 (tt, *J* = 11.8, 3.4 Hz, 1H), 1.97 (dd, *J* = 13.5, 3.6 Hz, 2H), 1.86 (dt, *J* = 12.7, 3.3 Hz, 2H), 1.78–1.73 (m, 1H), 1.56 (dd, *J* = 12.5, 3.2 Hz, 1H), 1.50 (dd, *J* = 12.4, 3.1 Hz, 1H), 1.42 (dt, *J* = 12.4, 3.3 Hz, 1H), 1.36 (dt, *J* = 12.4, 3.1 Hz, 1H), 1.32–1.21 (m, 1H). ^13^C NMR (100 MHz, CDCl_3_) δ 174.7, 163.9, 157.2, 138.6, 138.2, 137.8, 135.4, 128.9, 127.72, 127.70, 127.5, 126.2, 124.0, 122.2, 115.9, 112.4, 101.2, 97.6, 46.0, 45.6, 32.0, 26.4, 26.1, 16.6. HRMS (APCI^−^) calcd for C_28_H_30_N_5_O_2_S (M–H)^−^ 500.2126, found 500.2128.

### 3.2. p97 Biochemical Assay

Purification of p97 was performed as described [55]. Reactions were conducted in duplicate in 96 well-half-area-UV-transparent microplates (Corning Inc., Corning, NY, USA) at a final volume of 100 µL. A master mix containing 0.028 mg/mL p97 enzyme, 0.05 mg/mL PNP, and 0.2 mM MesG (Berry & Associates, Saint Joseph, MO, USA) in an assay buffer (50 mM Tris pH 8.0, 150 mM KCl, 1 mM MgCl_2_, 15% glycerol, and 1mM DTT) was preincubated for 5 min at room temperature with 1% DMSO or compounds (0.10 or 1.0 µM). Reactions were initiated by the addition of 0.2 mM ATP. The plate was mixed on an Eppendorf MixMate for 30 s at 1000 RPM (Eppendorf, Hamburg, Germany) and then monitored continuously at A_360_ on an i3 multi-mode plate reader (Molecular Devices, San Jose, CA, USA) set at 37 °C. The maximum slope of the linear portion of each reaction was used as its steady-state velocity to account for the initial lag in activity consistently observed for p97. Percentages of inhibition were calculated using GraphPad Prism version 10 (GraphPad Software; San Diego, CA, USA).

### 3.3. Cells and Viruses

A549/ACE2/TMPRSS2 cells [59] (provided by M. Saeed, Boston University; Boston, MA, USA) were maintained in Dulbecco’s modified Eagle’s medium (DMEM) high glucose supplemented with 10% fetal bovine serum (FBS), 100 IU streptomycin/penicillin per mL, 10 mM HEPES, 1× Non-Essential Amino Acids (NEAA), 1× GlutaMAX, 1mM sodium pyruvate, 5 µg/mL plasmocin, 0.5 µg/mL puromycin and 0.5 µg/mL blasticidin. SARS-CoV-2 isolate USA-WA1/2020 (NR-52281) was obtained through BEI Resources (Manassas, VA, USA) and propagated in Vero-E6 cells.

### 3.4. SARS-CoV-2 Immunofluorescence Antiviral Assay

The immunofluorescence antiviral assay was performed as described [64]. Briefly, 1.5 × 10^4^ A549/ACE2/TMPRSS2 cells per well were plated in 96 well plates and the next day cells were incubated in a compound plus infection medium (DMEM high glucose supplemented with 5% FBS, 100 IU streptomycin/penicillin per mL, 10 mM HEPES, 1× NEAA, 1× GlutaMAX and 1mM sodium pyruvate) prior to inoculation with SARS-CoV-2 isolate USA-WA1/2020 (NR-52281) at a multiplicity of infection of 0.01. The final DMSO concentration was 0.5%. After forty-eight hours, the cells were fixed with 4% PFA in 1× PBS for 30 min and incubated with an anti-SARS-CoV-2 nucleoprotein antibody (Sino Biologicals 40588-T62; Wayne, PA, USA) followed by a fluorescent secondary antibody as described [65]. The total numbers of DAPI-positive cells and fluorescing (infected) cells in each well were determined using a BioTek Cytation 1^TM^ imaging reader (Winooski, VT, USA). The percentage of infected cells was determined by dividing the number of infected cells by the total number of cells. The percentage of infected cells and the total number of cells for each treatment was plotted into GraphPad Prism version 10 to perform a non-linear regression analysis, to generate infectious dose–response curves, and to calculate EC_50_ values.

### 3.5. Cell Viability Assay

Viability assays in non-infected A549/ACE2/TMPRSS2 cells were performed using the 3-(4,5-dimethylthiazol-2-yl)-5-(3-carboxymethoxyphenyl)-2-(4-sulfophenyl)-2H-tetrazolium (MTS)-based tetrazolium reduction CellTiter 96 Aqueous Non-Radioactive cell proliferation assay (Promega G5430; Madison, WI, USA) as described [64]. Assays were conducted in parallel to the immunofluorescence antiviral assay. Experimental details, including the cell line used, number of cells seeded into plates, and compound incubation time, corresponded to those used in the immunofluorescence antiviral assay above. The data obtained were used to calculate the CC_50_ value using GraphPad Prism software.

### 3.6. Molecular Docking

The cryo-EM structure of human p97 (PDB 5FTK) [49] was downloaded from the Protein Data Bank.

After the protein structure was processed, a receptor grid was created to encompass the active site of the D2 domain at the interface of subunits D and E as described [55]. The molecular docking was performed using the Schrodinger modeling package (version 2024-3). The p97 inhibitors were created and processed using the LigPrep application to generate conformation and ionization states within a pH of 7 ± 2 (Epik) in Maestro version 14.1.13. The docking of p97 ligands into the receptor grid was carried out using the standard Glide XP mode. Covalent docking was performed using the CovDock [60] application and Cys522 was designated as the nucleophilic residue, which was pre-set to undergo a conjugate addition to an alkyne (carbonyl activated). Structural visualization and representation were performed with PyMOL version 2.5.0.

## 4. Conclusions and Perspectives

In short, we have successfully designed and synthesized new p97 inhibitors by rearranging the central fused ring of our previously reported p97 inhibitors. Among these compounds, **8a**, **9a**, and **10a** possess enhanced anti-p97 activity over their parent compounds. More importantly, compounds **9a** and **10a** exhibit good antiviral activity against SARS-CoV-2 without significant cytotoxicity. Molecular docking reveals no major perturbation of the binding mode relative to that of their parent compounds, further supporting our design strategy. Taken together, compounds **9a** and **10a** are new, nontoxic p97 inhibitors that possess promising antiviral activity against SARS-CoV-2. Since p97 is implicated in the replication of many important viral pathogens, we anticipate that these p97 inhibitors may be effective against viruses besides SARS-CoV-2. Therefore, further SAR studies and antiviral evaluation are justified. Nonetheless, multiple important questions remain to be answered. What structural features and subsequent binding modes contribute to the antiviral activity and low cytotoxicity? What p97-associated cellular functions are affected by these compounds? Which of these cellular functions are key for viral replication? Insights into the cellular effects of these compounds in the context of viral replication in combination with the availability of improved, nontoxic p97 inhibitors will certainly allow us to further explore p97 as a promising host antiviral target.

## Figures and Tables

**Table 1:** Evaluation of p97 inhibitors.

		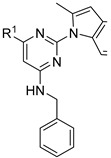					
Cmpd	R^1^	R^2^	p97 Inhib. (%) at 0.10/1.0 µM ^a^	SARS-CoV-2			CC_50_ (µM)
				Conc. (µM)	Inhib. (%) ^a^	EC_50_ (µM) ^a^	
CB-5083			62/92	0.38	6 ± 4	NT ^c^	0.95
NMS-873			5/84	1.6	0 ± 2	NT ^c^	4.1
**6a**			57/97	0.22	6 ± 3	NT ^c^	0.55
**6b**			38/95	0.46	5 ± 0	NT ^c^	1.2
**8a**	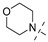	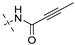	86/97	0.96	3 ± 5	NT ^c^	2.4
**8b**	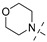	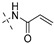	77/96	2.1	5 ± 8	NT ^c^	5.2
**8c**	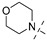	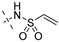	65/89	2.1	8 ± 3	NT ^c^	5.2
**9a**	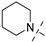	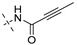	94/100	30	98 ± 0	6.7 ± 0.8	76
**9b**	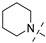	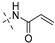	64/91	50	28 ± 3	NT ^c^	ND ^d^
**9c**	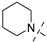	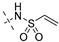	73/95	5.9	0 ± 4	NT ^c^	15
**10a**	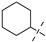	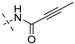	88/100	50	96 ± 1	6.8 ± 0.6	>100
**10b**	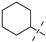	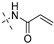	70/96	50	30 ± 9	NT ^c^	>100
**10c**	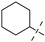	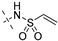	68/95	4.8	7 ± 3	NT ^c^	12
**11**	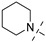	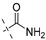	0/0	NA ^b^	NT ^c^	NT ^c^	NT ^c^
**12**	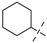	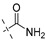	18/21	NA ^b^	NT ^c^	NT ^c^	NT ^c^
remdesivir			NA ^b^	2.0	100 ± 0	0.044	>10

^a^ Experiments performed at least twice with the mean and standard error listed. ^b^ NA, not applicable. ^c^ NT, not tested. ^d^ ND, not determined, cytostatic.

## Data Availability

Data are contained within the article and the Supplementary Materials. Further inquiries can be directed to the corresponding author.

## Supplementary Materials

The following supporting information can be downloaded at: https://www.mdpi.com/article/10.3390/ph18010131/s1, Chemical synthesis of compounds **11** and **12**, Antiviral dose-response curves for compounds **9a** and **10a**, and ^1^H NMR, ^13^C NMR, and HRMS spectra for final compounds.
