# Supplementary material for: p97 Inhibitors Possessing Antiviral Activity Against SARS-CoV-2 and Low Cytotoxicity
Source: Pharmaceuticals (Basel). 2025 Jan 19;18(1):131. doi: 10.3390/ph18010131 (PMC11768289; doi:10.3390/ph18010131)
Supplement: Supplementary file 1 [file pharmaceuticals-18-00131-s001.zip › pharmaceuticals-3382140-supplementary.pdf]

Supplementary Materials

p97 Inhibitors Possessing Antiviral Activity Against SARS-CoV-2 and Low Cytotoxicity

Rui Ding[1], Tiffany C. Edwards[1], Prithwish Goswami[1], Daniel J. Wilson[1], Christine D. Dreis[1], Yihong Ye[2], Robert J. Geraghty[1,*] and Liqiang Chen[1,*]

[1] Center for Drug Design, College of Pharmacy, University of Minnesota, Minneapolis, MN 55455, USA
[2] Laboratory of Molecular Biology, National Institute of Diabetes and Digestive and Kidney Diseases, National Institutes of Health, Bethesda, MD 20892, USA

Supplementary Materials and Methods

*3.1.20. tert-Butyl benzyl(6-chloro-2-(methylsulfonyl)pyrimidin-4-yl)carbamate (**27**).* A solution of **25** [1] (4.46 g, 16.8 mmol), Boc_2O (4.53 g, 20.8 mmol), and DMAP (12 mg, 0.10 mmol) in anhydrous THF (50 mL) was allowed to stir at rt for 16 h. After the organic solvent was removed in vacuo, the crude **26** was used for next reaction without purification. To the residue in CH_2Cl_2 (100 mL) at 0 °C was added mCPBA (55% purity, 11.7 g, 37.3 mmol) and the mixture was allowed to warm to rt and stir at rt for 3 h. The reaction was quenched with saturated $NaHCO_3$ and the mixture was extracted with CH_2Cl_2. The organic layer was concentrated and the residue was purified by flash column chromatography (30% EtOAc/hexanes) to give compound **27** as a white solid (6.39 g, 96% for 2 steps). ^{1}H NMR (400 MHz, $CDCl_3$) δ 8.37 (s, 1H), 7.30 (d, *J* = 4.4 Hz, 4H), 7.26–7.23 (m, 1H), 5.30 (s, 2H), 3.16 (s, 3H), 1.51 (s, 9H). ^{13}C NMR (100 MHz, $CDCl_3$) δ 164.5, 162.6, 162.0, 152.7, 137.1, 128.7, 127.7, 127.5, 114.3, 85.0, 49.5, 39.1, 28.1. HRMS (ESI$^+$) calcd for $C_{17}H_{21}ClN_3O_4S$ $(M+H)^+$ 398.0936, found 398.0940.

*3.1.21. 2-Methyl-1H-indole-4-carboxamide (**29**).* A solution of 2-methyl-1*H*-indole-4-carboxylic acid (**28** [2], 1.16 g, 6.63 mmol), Boc2O (5.71 g, 26.2 mmol), Et_3N (2.0 mL, 14.3 mmol), and DMAP (7.1 mg, 0.058 mmol) in anhydrous THF (30 mL) was allowed to stir at rt for 45 min and 7N NH_3 in MeOH (10 mL) was added. The resulting white suspension was allowed to stir for additional 25 min and concentrated. The residue was purified by flash column chromatography ($MeOH/CH_2Cl_2$ = 10:1) to give compound **29** as a yellow solid (632 mg, 54%). ^{1}H NMR (400 MHz, CD_3OD) δ 7.42 (dd, *J* = 7.8, 1.2 Hz, 2H), 7.06 (td, *J* = 7.8, 1.1 Hz, 1H), 6.57 (s, 1H), 2.45 (s, 3H).^{13}C NMR (100 MHz, CD_3OD) δ 174.6, 138.9, 138.8, 128.7, 125.4, 120.5, 120.4, 114.9, 100.8, 13.5. HRMS (ESI$^-$) calcd for $C_{10}H_9N_2O$ $(M-H)^-$ 173.0720, found 173.0721.

*3.1.22. tert-Butyl benzyl(2-(4-carbamoyl-2-methyl-1H-indol-1-yl)-6-chloropyrimidin-4-yl)carbamate (**30**).* A solution of 2-methyl-1*H*-indole-4-carboxamide (**29**, 210 mg, 1.21 mmol) and LDA (1 N in THF, 3.0 mL) in anhydrous THF (20 mL) was allowed to stir at rt for 15 min and **27** (820 mg, 2.06 mmol) was added. The resulting red solution was allowed to stir at rt for 40 min and additional LDA (1 N in THF, 1.0 mL) was added. The dark red solution was stirred for 30 min and quenched with saturated NH_4Cl. After the mixture was extracted with CH_2Cl_2, the organic layer was concentrated and the residue was purified by flash column chromatography (50% EtOAc/hexanes) to give compound **30** as a light yellow solid (280 mg, 47%). ^{1}H NMR (400 MHz, $CDCl_3$) δ 8.56 (brs, 1H), 8.20 (brs, 1H), 7.89 (s, 1H), 7.56 (dd, *J* = 7.5, 0.9 Hz, 1H), 7.45 (dt, *J* = 8.0, 0.9 Hz, 1H), 7.35–7.32 (m, 2H), 7.29–7.26 (m, 1H), 7.25–7.24 (m, 1H), 7.23–7.21 (m, 1H), 7.15 (t, *J* = 7.8 Hz, 1H), 6.70–6.69 (m, 1H), 5.33 (s, 2H), 2.47 (s, 3H), 1.46 (s, 9H). ^{13}C NMR (100 MHz, $CDCl_3$) δ 165.7, 162.1, 161.8, 156.4, 153.3, 138.6, 138.1, 137.0, 128.4, 127.8, 127.4, 127.2, 125.0, 120.6, 120.4, 114.5, 107.3, 100.7, 83.5, 48.8, 28.1, 14.0. HRMS (APCI$^+$) calcd for $C_{26}H_{27}ClN_5O_3$ $(M+H)^+$ 492.1797, found 492.1796.

*3.1.23. tert-Butyl benzyl(2-(4-carbamoyl-2-methyl-1H-indol-1-yl)-6-(piperidin-1-yl)pyrimidin-4-yl)carbamate (**11**).* A solution of **30** (29 mg, 0.059 mmol) and piperidine (30 μL, 0.30 mmol) in dioxane (1 mL) was heated at 100 °C for 1.5 h and the organic solvent was removed in vacuo. The resulting crude **31** was used directly for next reaction. The residue was dissolved in anhydrous CH_2Cl_2 (3 mL) and TFA (1 mL) was added. The mixture was allowed to stir at rt for 2 h, quenched

with saturated $NaHCO_3$, and concentrated. The residue was purified by flash column chromatography (EtOAc) to give compound **11** as a white solid (12 mg, 45% for 2 steps). ^{1}H NMR (400 MHz, $CDCl_3$) δ 8.28 (s, 2H), 7.56 (dd, *J* = 7.5, 0.8 Hz, 1H), 7.41 (d, *J* = 8.1 Hz, 1H), 7.33 (d, *J* = 4.6 Hz, 3H), 7.31–7.26 (m, 1H), 7.13 (t, *J* = 7.8 Hz, 1H), 6.75 (s, 1H), 5.25 (brs, 1H), 5.21 (s, 1H), 4.42 (d, *J* = 5.8 Hz, 2H), 3.49 (t, *J* = 5.4 Hz, 4H), 2.46 (s, 3H), 1.64–1.59 (m, 2H), 1.57–1.52 (m, 4H). ^{13}C NMR (100 MHz, $CDCl_3$) δ 166.5, 164.5, 163.8, 157.0, 138.6, 137.5, 137.0, 128.8 (2C), 127.54, 127.47, 127.3, 126.4, 120.5, 120.1, 113.8, 100.8, 46.3, 45.4, 25.6, 24.9, 14.0. HRMS (ESI^-) calcd for $C_{26}H_{27}N_6O$ $(M–H)^-$ 439.2252, found 439.2255.

*3.1.24. tert-Butyl benzyl(2-(4-carbamoyl-2-methyl-1H-indol-1-yl)-6-(cyclohex-1-en-1-yl)pyrimidin-4-yl)carbamate (**32**).* A mixture of **30** (70 mg, 0.14 mmol), cyclohex-1-en-1-ylboronic acid (23 mg, 0.18 mmol), $Pd(PPh_3)_2Cl_2$ (6 mg, 0.008 mmol), Na_2CO_3 (39 mg, 0.37 mmol) in dioxane (2 mL) and H_2O (1 mL) was heated at 100 °C for 1 h. After concentration, the residue was purified by flash column chromatography (30% EtOAc/hexanes) to give compound **32** as a white solid (65 mg, 85%). ^{1}H NMR (400 MHz, $CDCl_3$) δ 8.47 (s, 1H), 8.13 (brs, 1H), 7.74 (s, 1H), 7.58 (d, *J* = 7.4 Hz, 1H), 7.42 (d, *J* = 8.0 Hz, 1H), 7.33–7.30 (m, 2H), 7.27–7.26 (m, 1H), 7.25–7.23 (m, 1H), 7.21–7.19 (m, 1H), 7.15 (t, *J* = 7.8 Hz, 1H), 7.10– 7.08 (m, 1H), 6.70–6.69 (m, 1H), 5.29 (s, 2H), 2.48–2.45 (m, 5H), 2.30–2.25 (m, 2H), 1.80–1.74 (m, 2H), 1.69–1.63 (m, 2H), 1.45 (s, 9H). ^{13}C NMR (100 MHz, $CDCl_3$) δ 167.4, 166.3, 162.0, 156.4, 153.7, 139.3, 137.7, 137.0, 135.1, 133.0, 128.3, 127.7, 127.4, 126.9, 126.0, 120.6, 120.3, 114.0, 103.3, 100.7, 82.6, 48.8, 28.3, 26.2, 25.5, 22.7, 22.1, 14.0. HRMS (ESI^+) calcd for $C_{32}H_{36}N_5O_3$ $(M+H)^+$ 538.2813, found 538.2817.

*3.1.25. 1-(4-(Benzylamino)-6-cyclohexylpyrimidin-2-yl)-2-methyl-1H-indole-4-carboxamide (**12**).* A mixture of **32** (38 mg, 0.071 mmol), 10 % Pd/C (4 mg) in MeOH (4 mL) was hydrogenated (balloon) at rt for 5 h. After filtration, the filtrate was concentrated and the obtained **33** was treated with 10% TFA/CH_2Cl_2 for 4 h. After the reaction was quenched with sat $NaHCO_3$, the mixture was extracted with CH_2Cl_2. The organic layer was concentrated and the residue was purified by flash column chromatography (10% $MeOH/CH_2Cl_2$) to give compound **12** as a white solid (19 mg, 85%). ^{1}H NMR (400 MHz, $CDCl_3$) δ 8.64 (s, 1H), 8.48 (brs, 1H), 7.57 (d, *J* = 7.5 Hz, 1H), 7.41 (d, *J* = 8.1 Hz, 1H), 7.35–7.33 (m, 1H), 7.31–7.30 (m, 3H), 7.29–7.27 (m, 1H), 7.10 (t, *J* = 7.8 Hz, 1H), 6.73 (s, 1H), 5.92 (s, 1H), 5.45 (brs, 1H), 4.48 (d, *J* = 5.7 Hz, 2H), 2.44 (s, 3H), 2.42–2.38 (m, 1H), 1.91–1.88 (m, 2H), 1.82–1.78 (m, 2H), 1.73–1.68 (m, 1H), 1.44–1.40 (m, 1H), 1.37–1.34 (m, 2H), 1.32–1.28 (m, 1H), 1.26–1.19 (m, 1H). ^{13}C NMR (100 MHz, $CDCl_3$) δ 175.1, 166.5, 164.1, 157.5, 138.1, 137.8, 137.0, 128.8, 127.7, 127.6, 127.4, 125.8, 120.3, 120.1, 114.2, 100.8, 95.3, 45.9, 32.1, 26.4 (2C), 26.1, 14.0. HRMS (ESI^+) calcd for $C_{27}H_{30}N_5O$ $(M+H)^+$ 440.2445, found 440.2434.

References

1. Cervi, G.; Magnaghi, P.; Asa, D.; Avanzi, N.; Badari, A.; Borghi, D.; Caruso, M.; Cirla, A.; Cozzi, L.; Felder, E.; et al. Discovery of 2-(cyclohexylmethylamino)pyrimidines as a new class of reversible valosine containing protein inhibitors. *J. Med. Chem.* **2014**, *57*, 10443-10454, doi:10.1021/jm501313x.
2. Torisu, K.; Kobayashi, K.; Iwahashi, M.; Nakai, Y.; Onoda, T.; Nagase, T.; Sugimoto, I.; Okada, Y.; Matsumoto, R.; Nanbu, F.; et al. Development of prostaglandin D2 receptor

antagonist: discovery of highly potent antagonists. *Bioorg. Med. Chem.* **2004**, *12*, 4685-4700, doi:10.1016/j.bmc.2004.06.024.

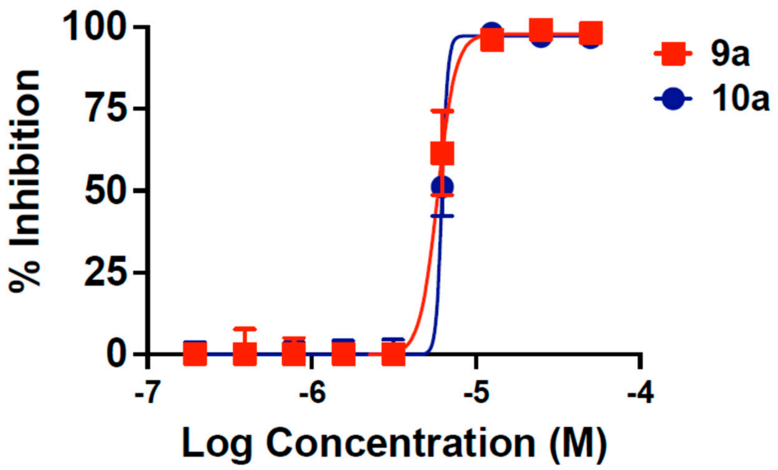

Figure S1. Dose-response curves of compounds **9a** and **10a** against SARS-CoV-2.

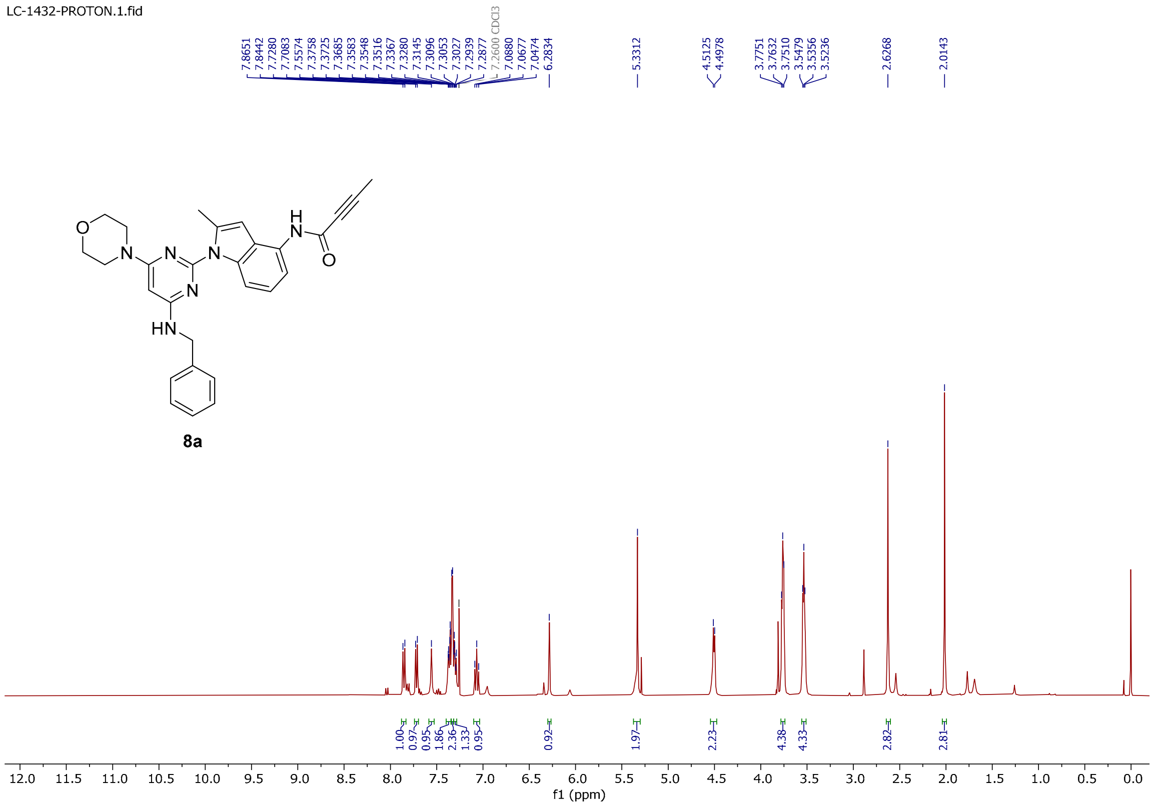

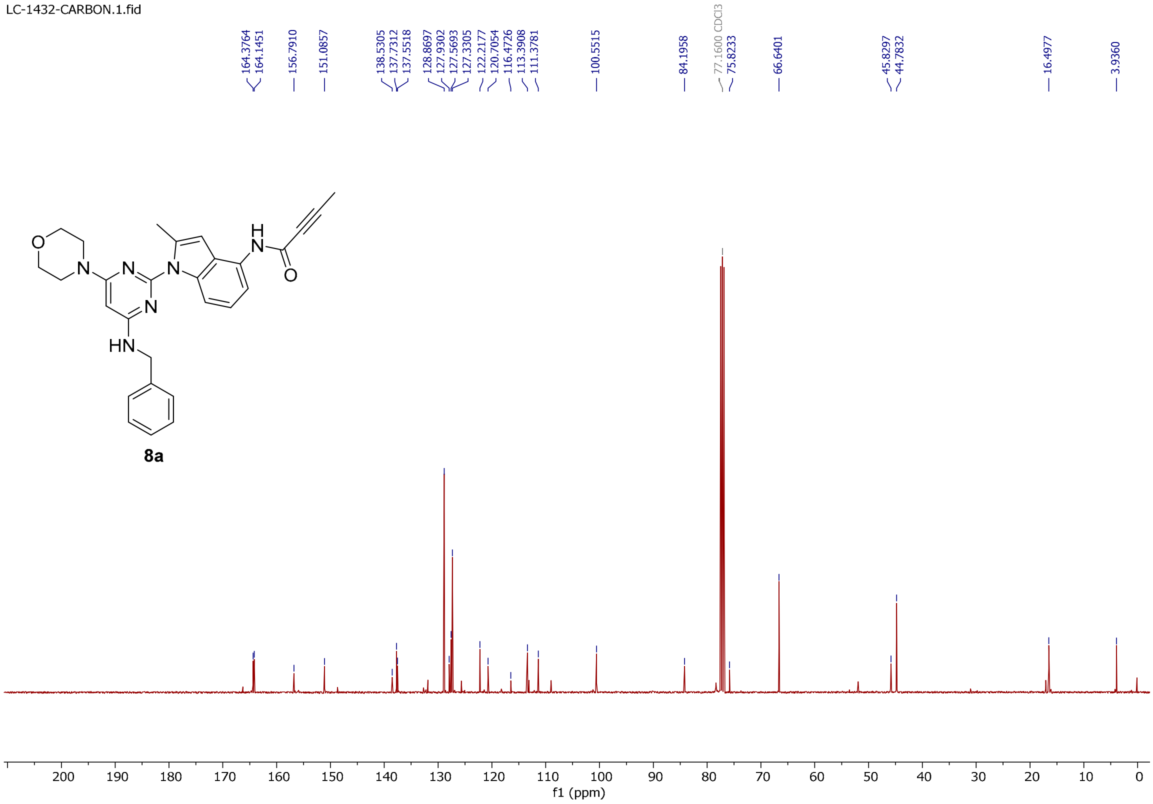

^{1}H and ^{13}C NMR spectra of compound **8a**

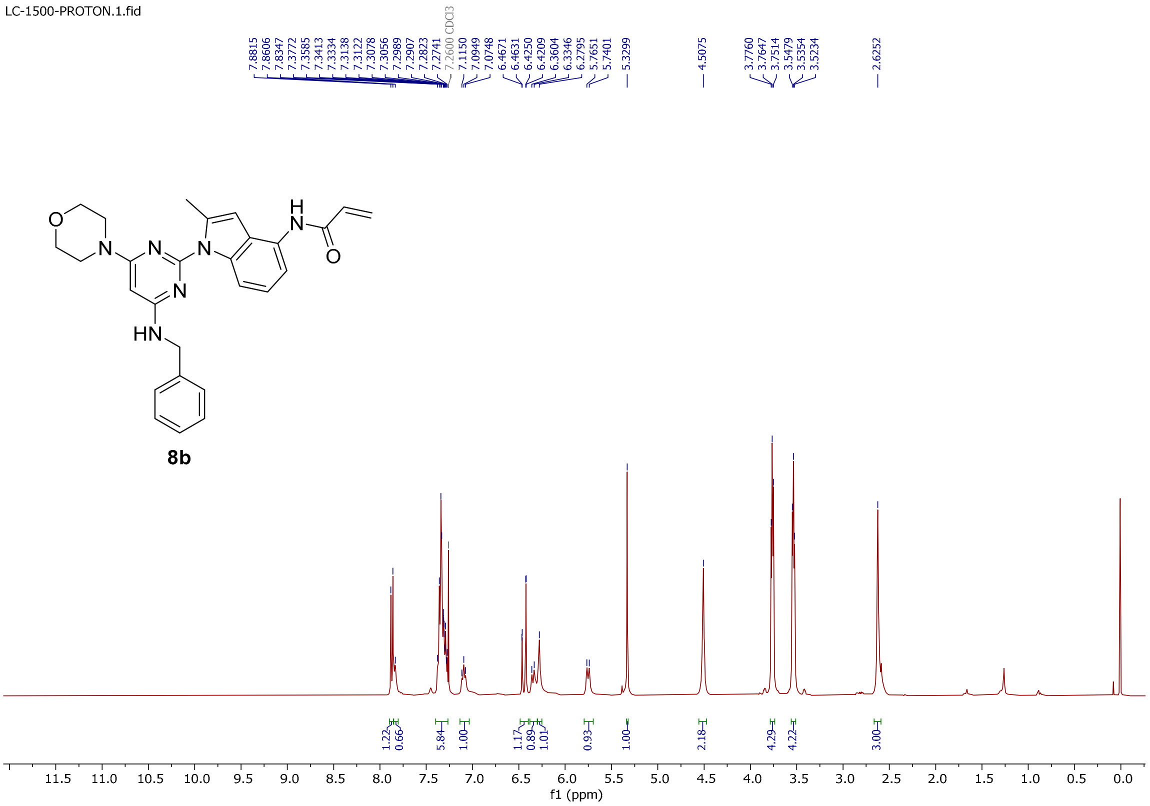

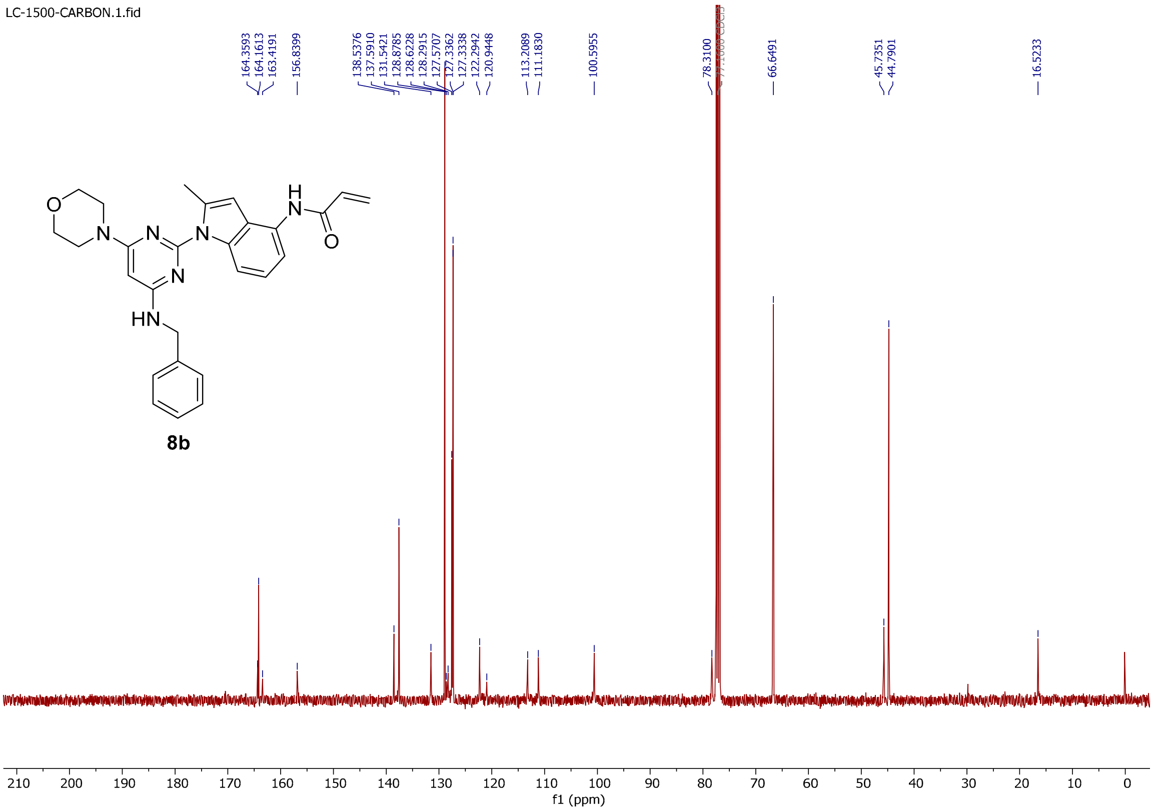

^{1}H and ^{13}C NMR spectra of compound **8b**

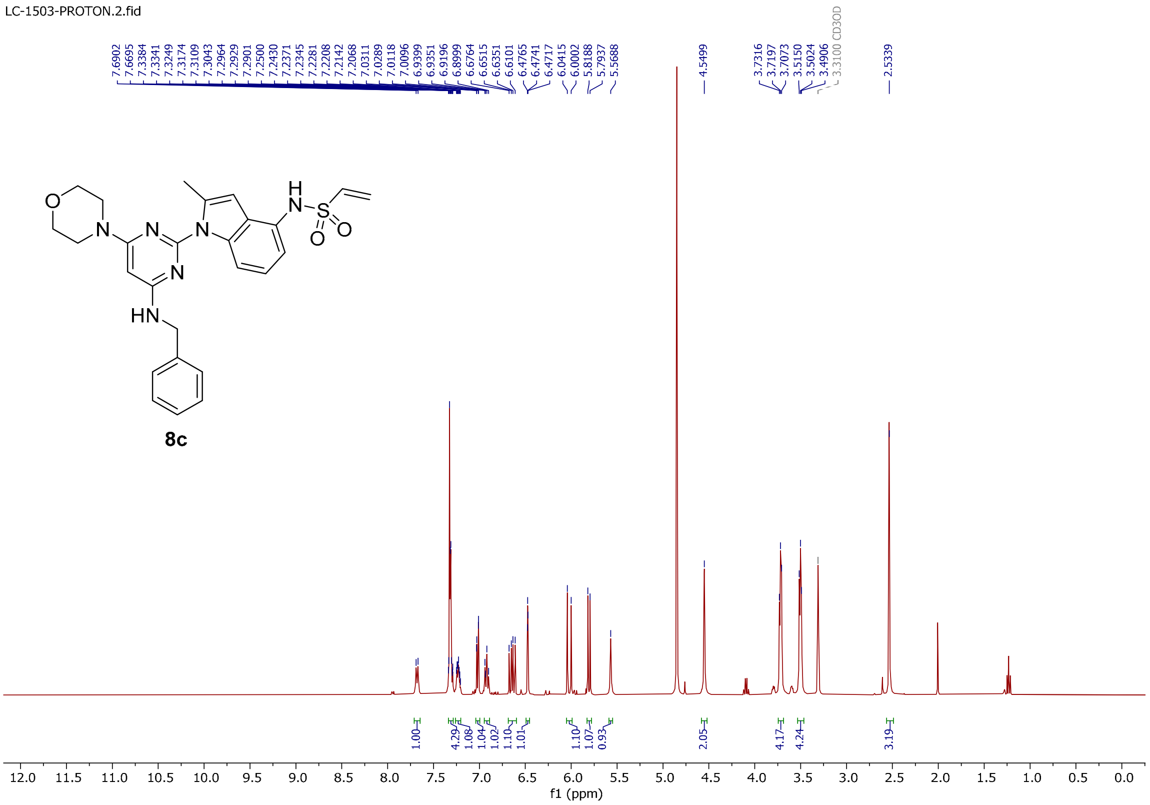

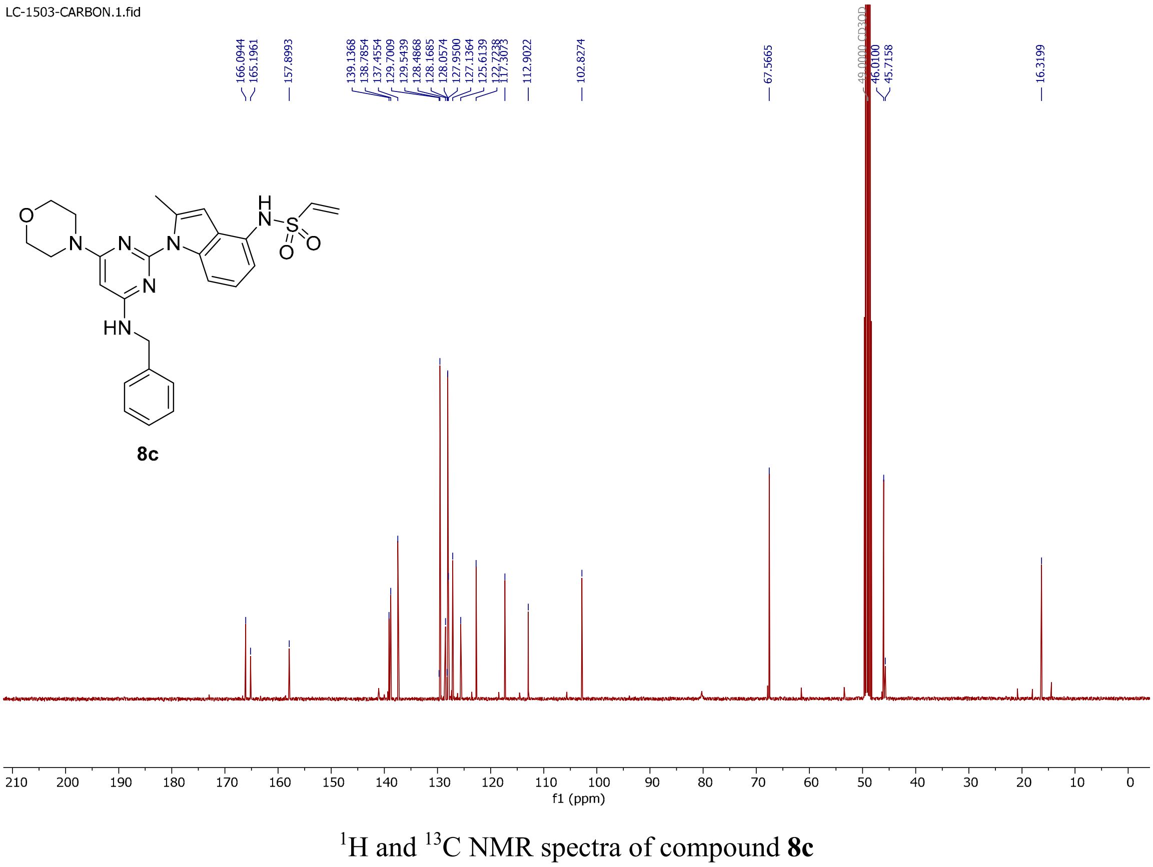

^{1}H and ^{13}C NMR spectra of compound **8c**

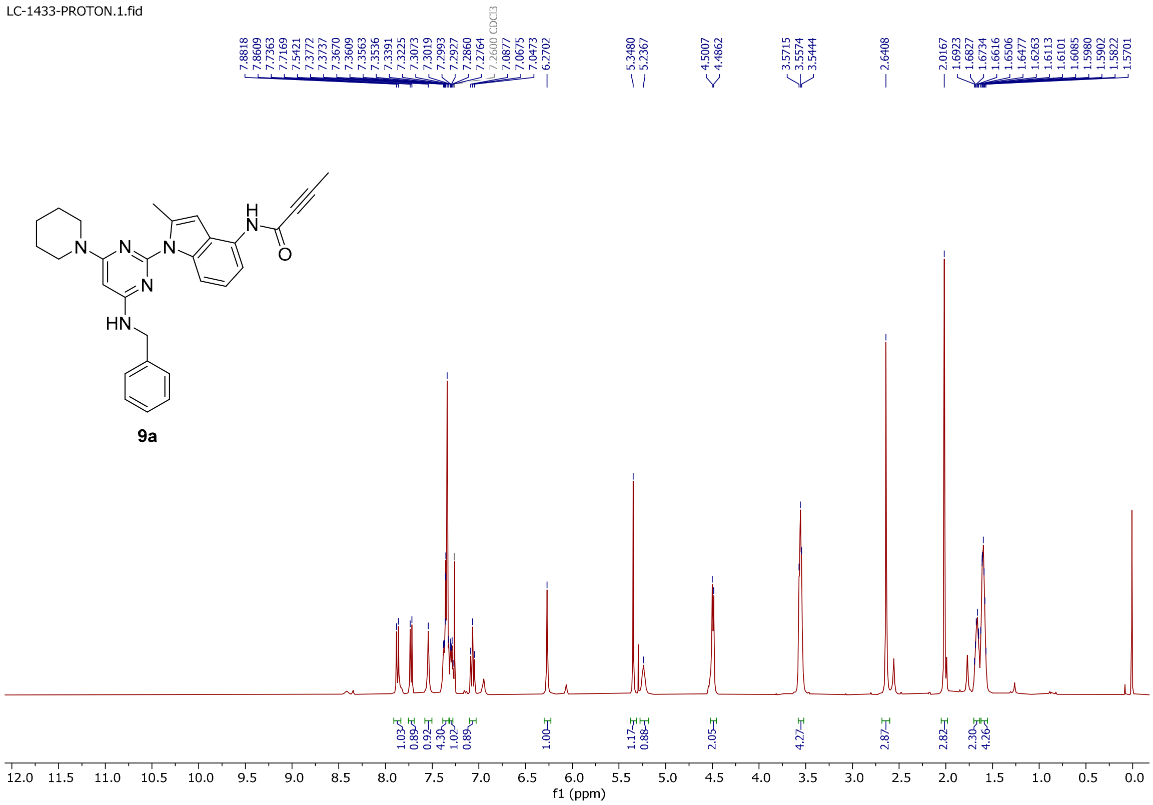

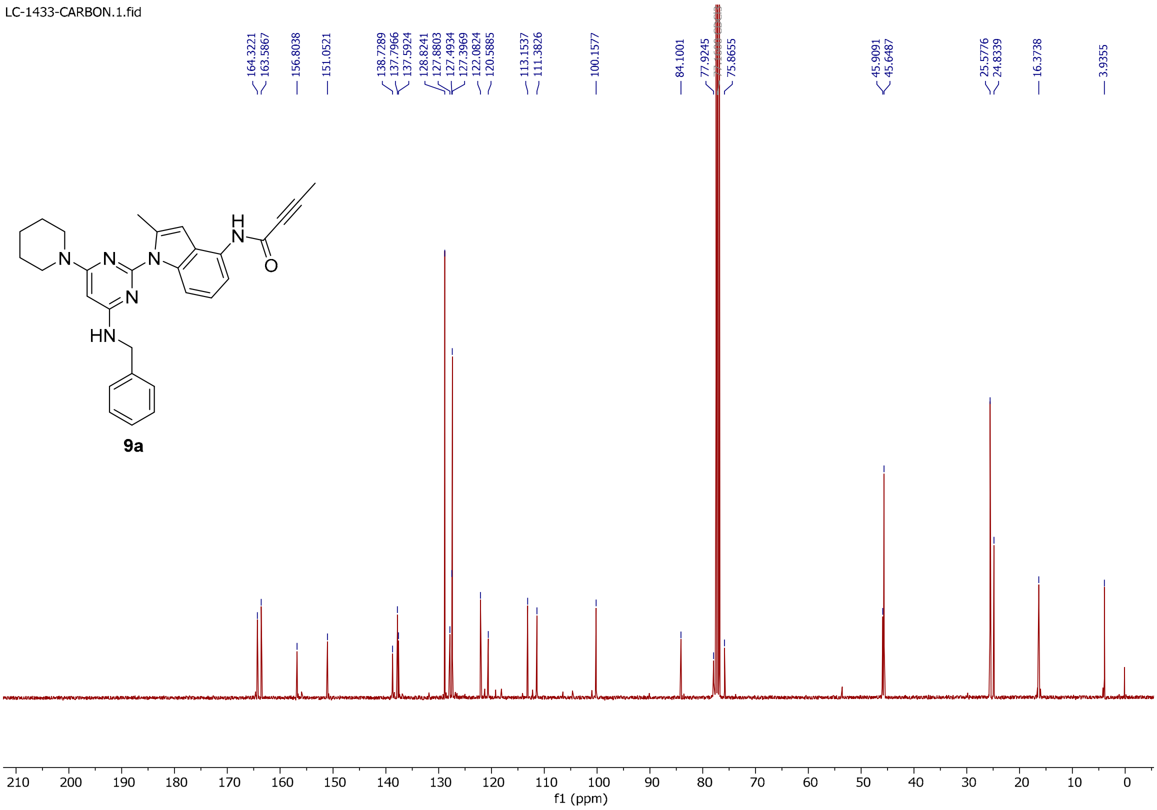

^{1}H and ^{13}C NMR spectra of compound **9a**

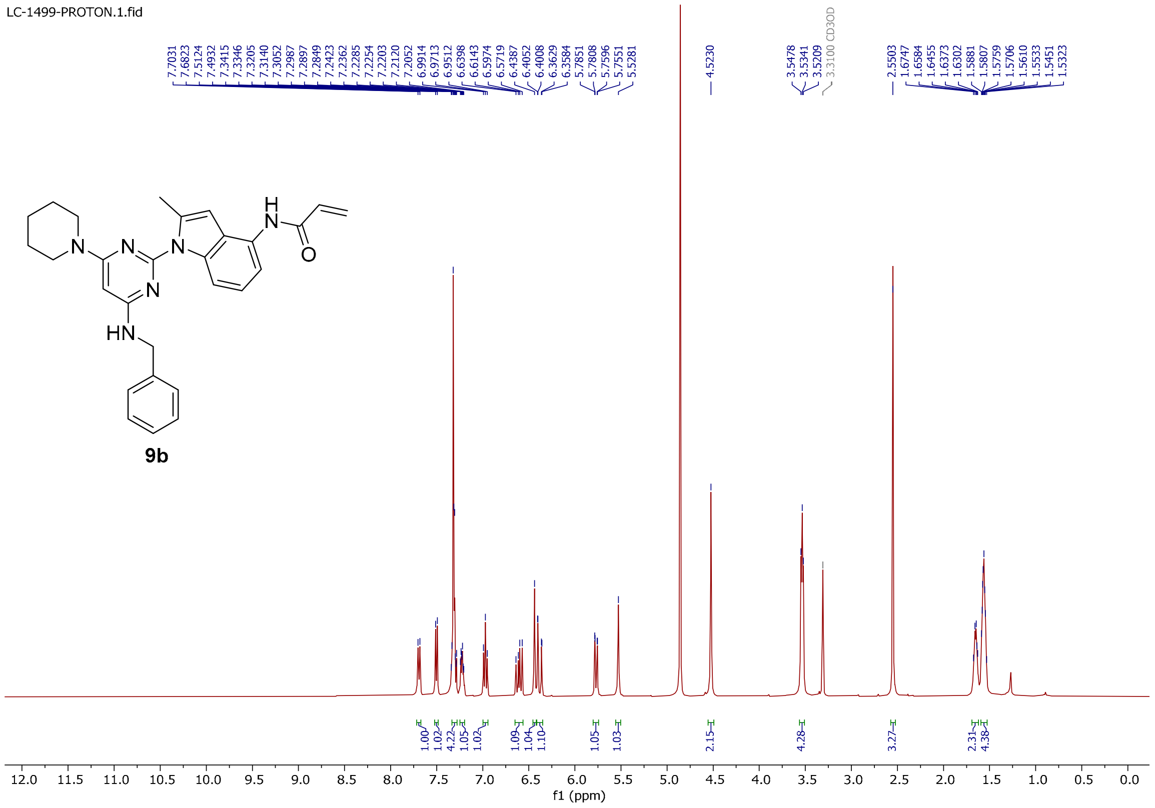

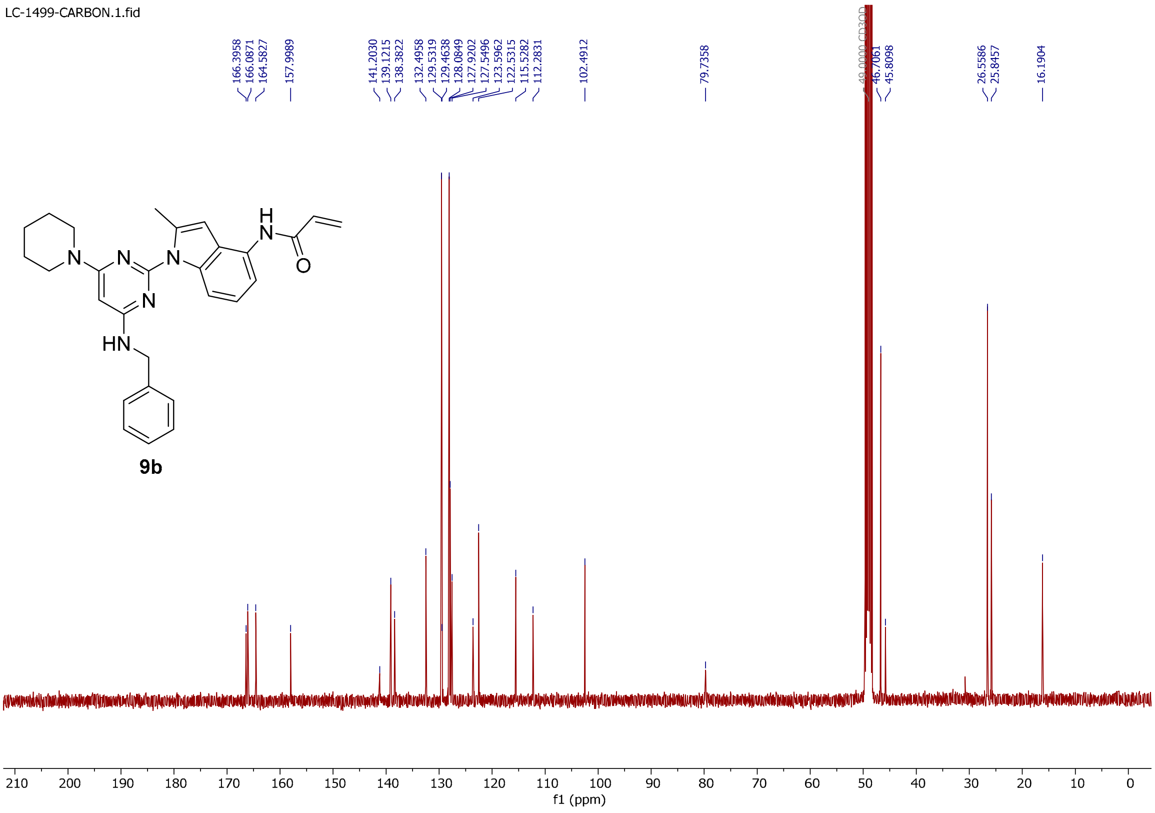

^{1}H and ^{13}C NMR spectra of compound **9b**

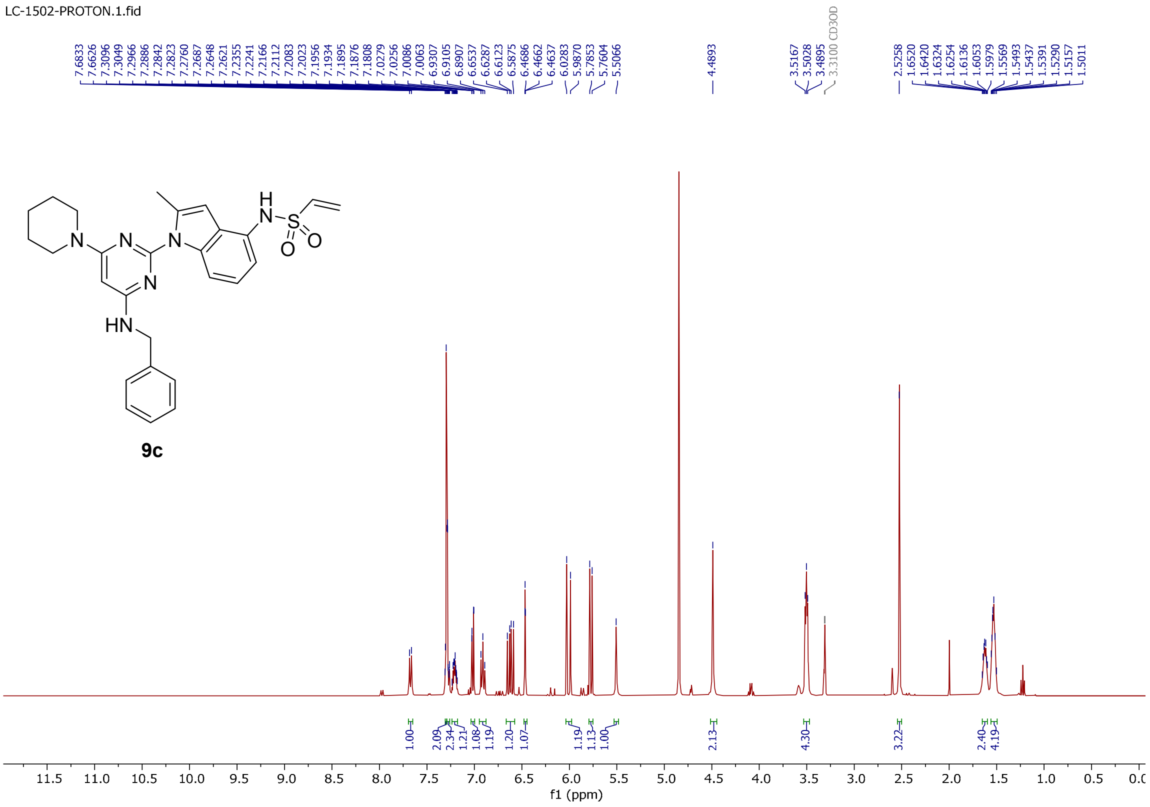

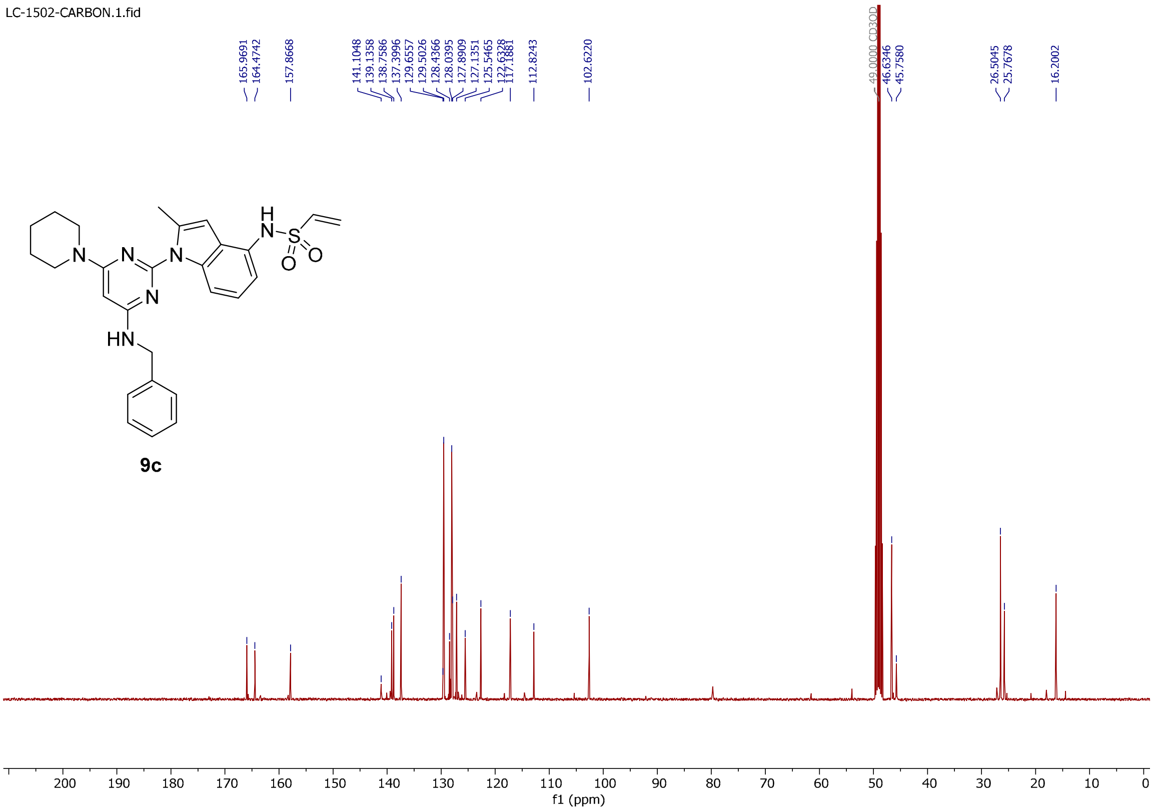

^{1}H and ^{13}C NMR spectra of compound **9c**

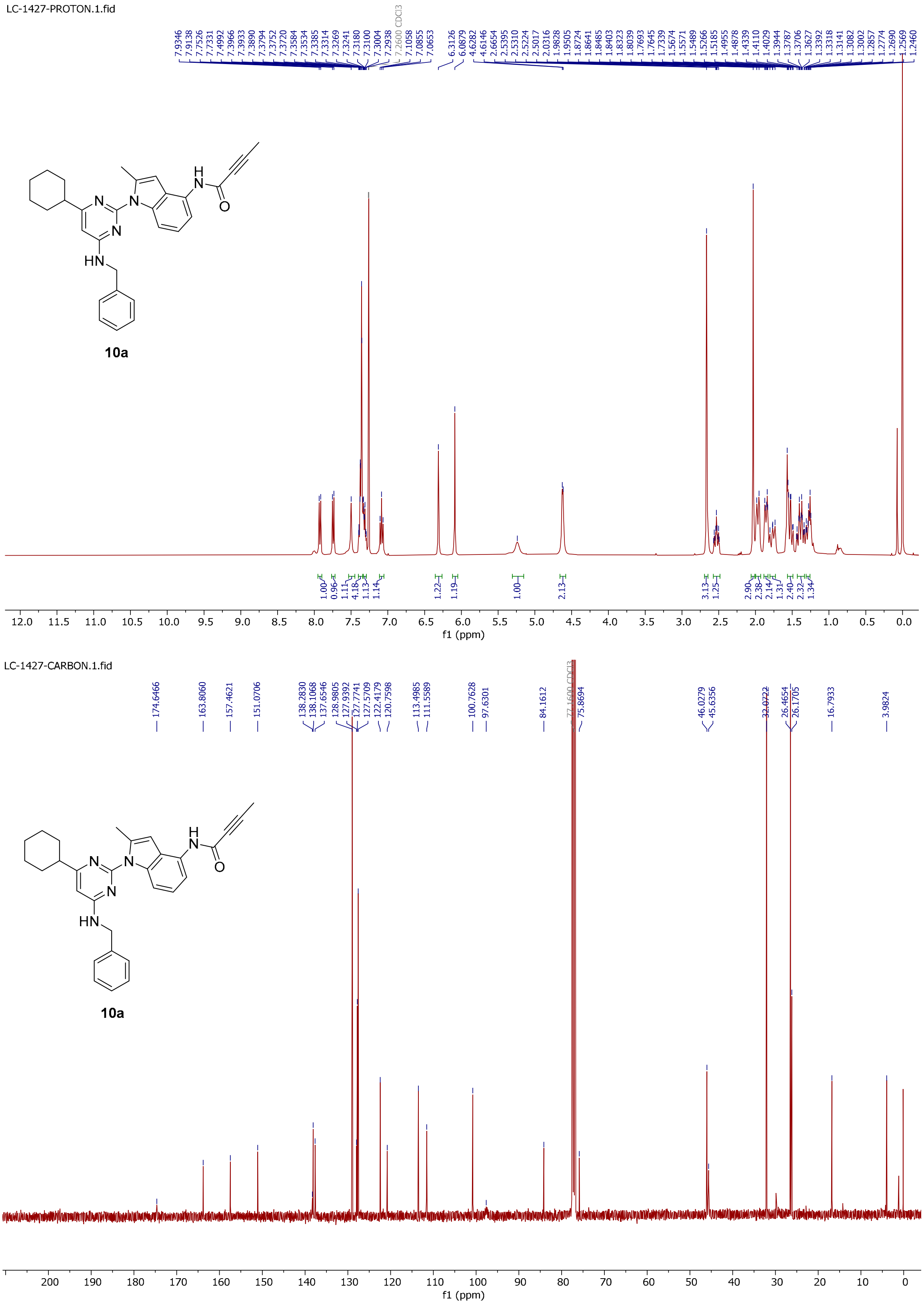

^{1}H and ^{13}C NMR spectra of compound **10a**

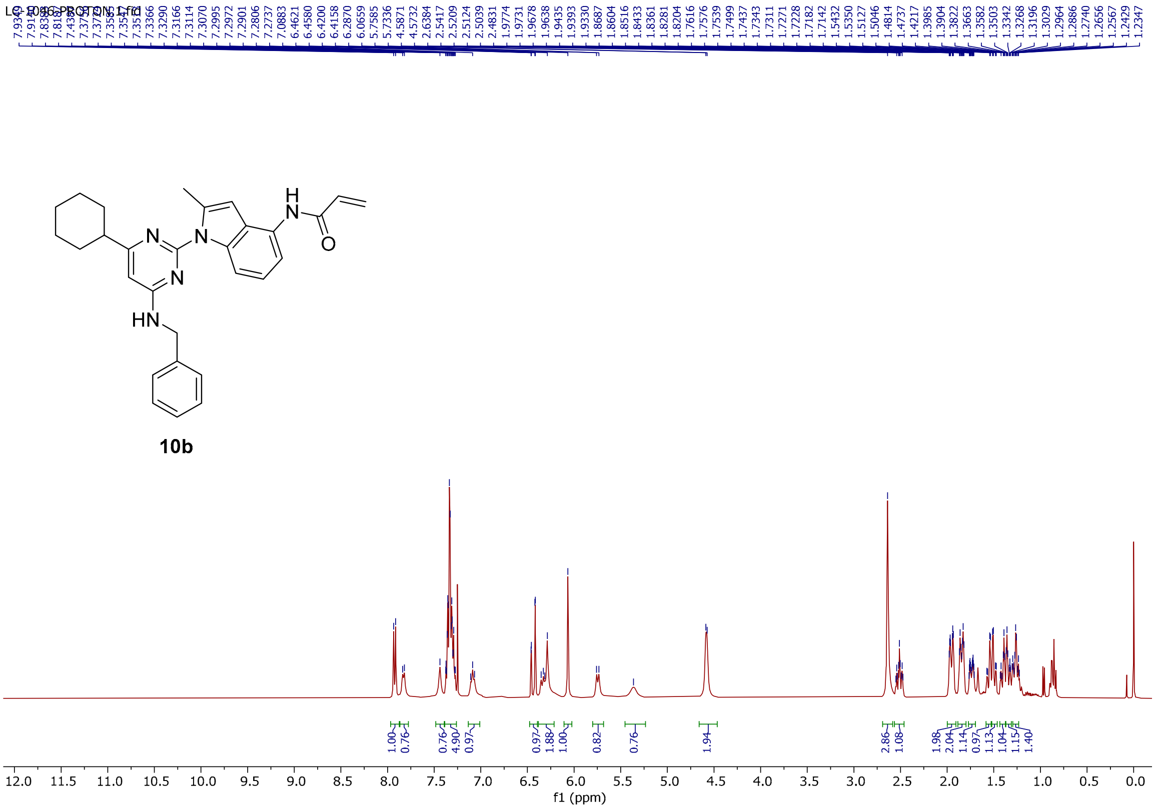

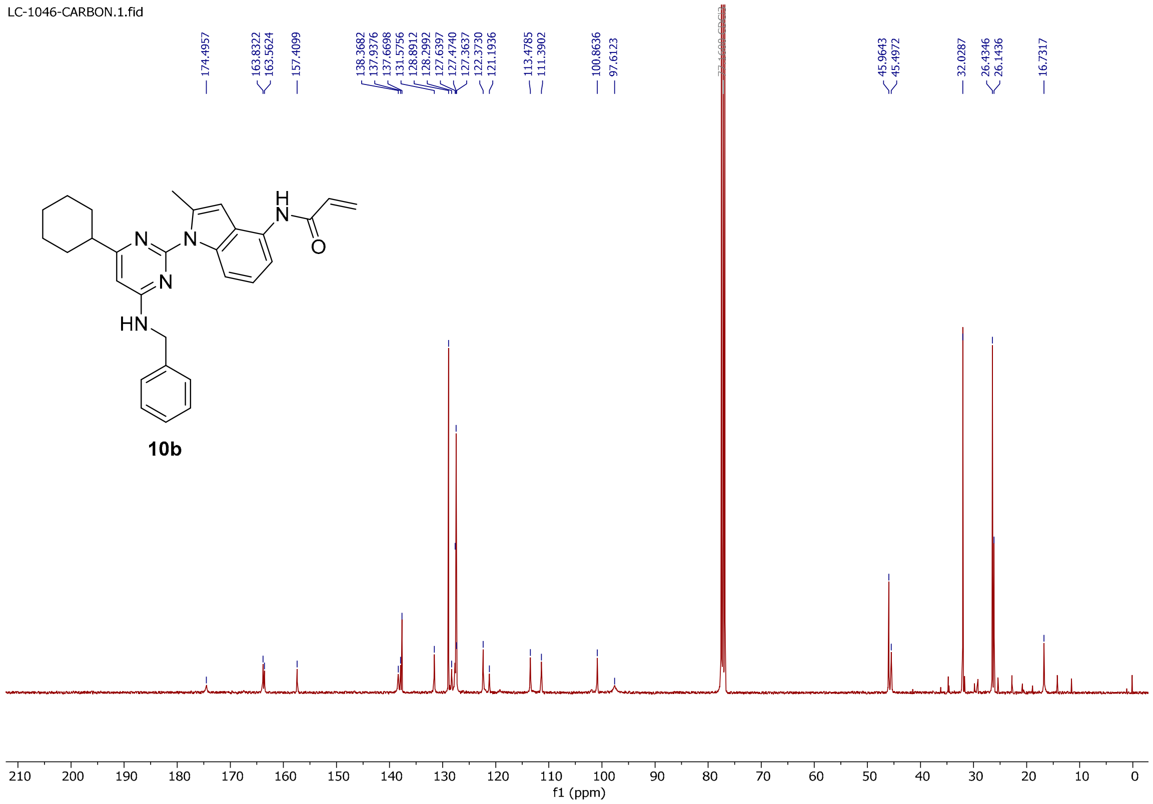

^{1}H and ^{13}C NMR spectra of compound **10b**

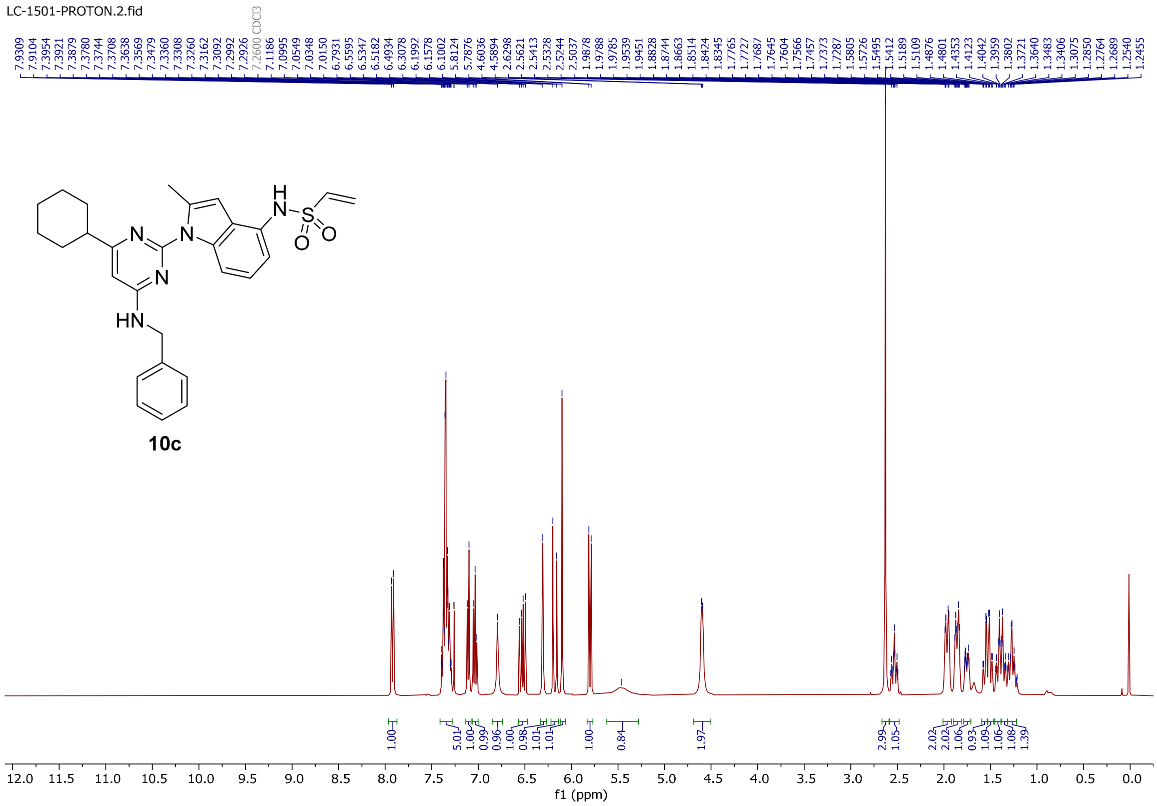

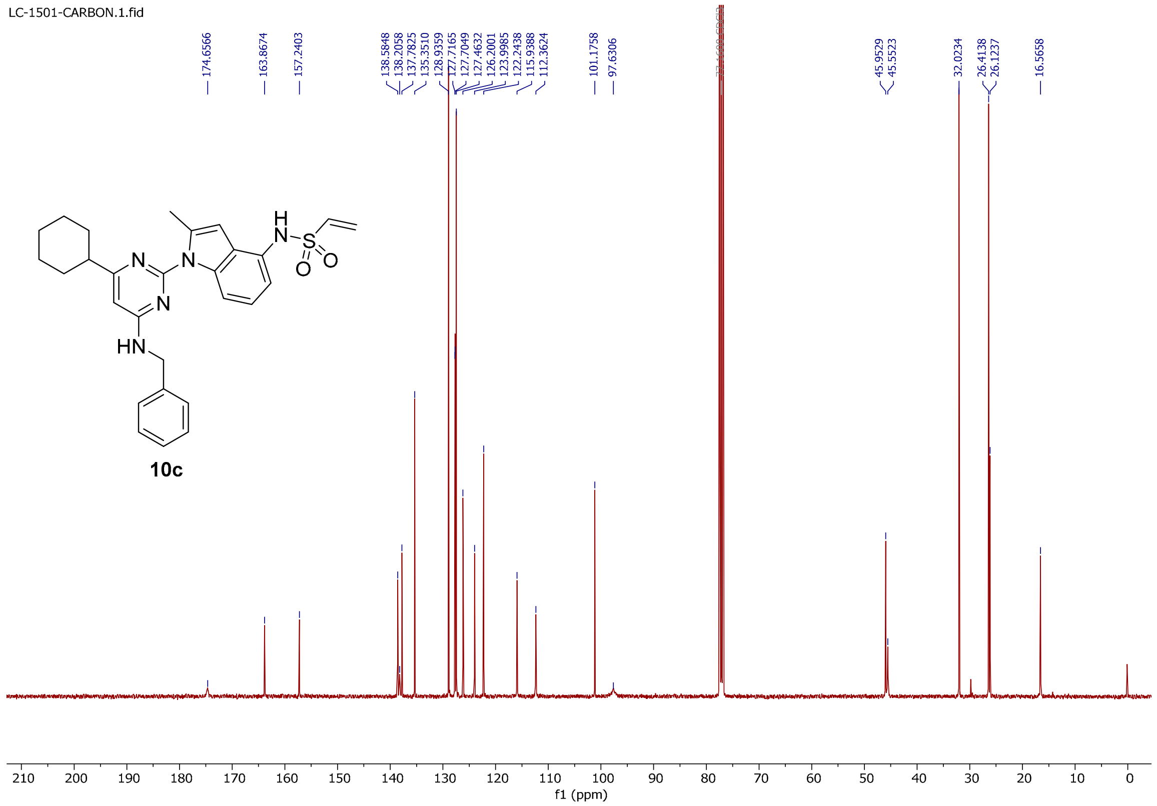

^{1}H and ^{13}C NMR spectra of compound **10c**

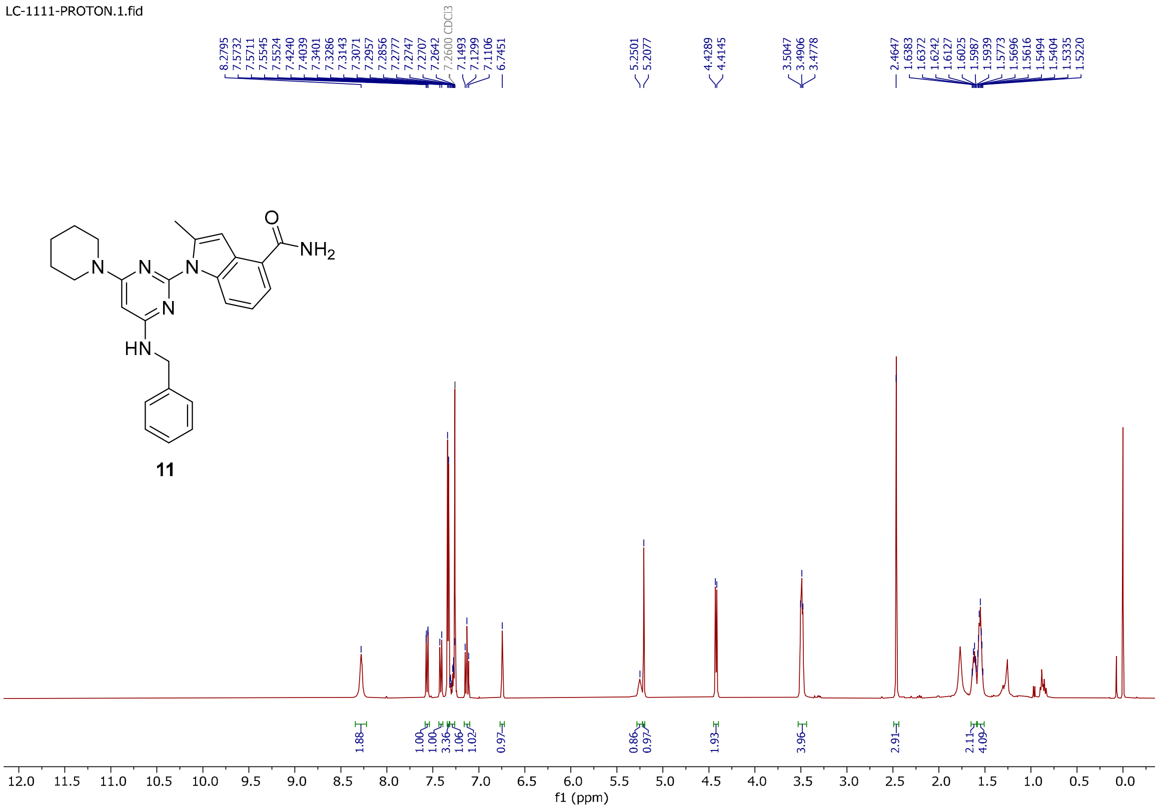

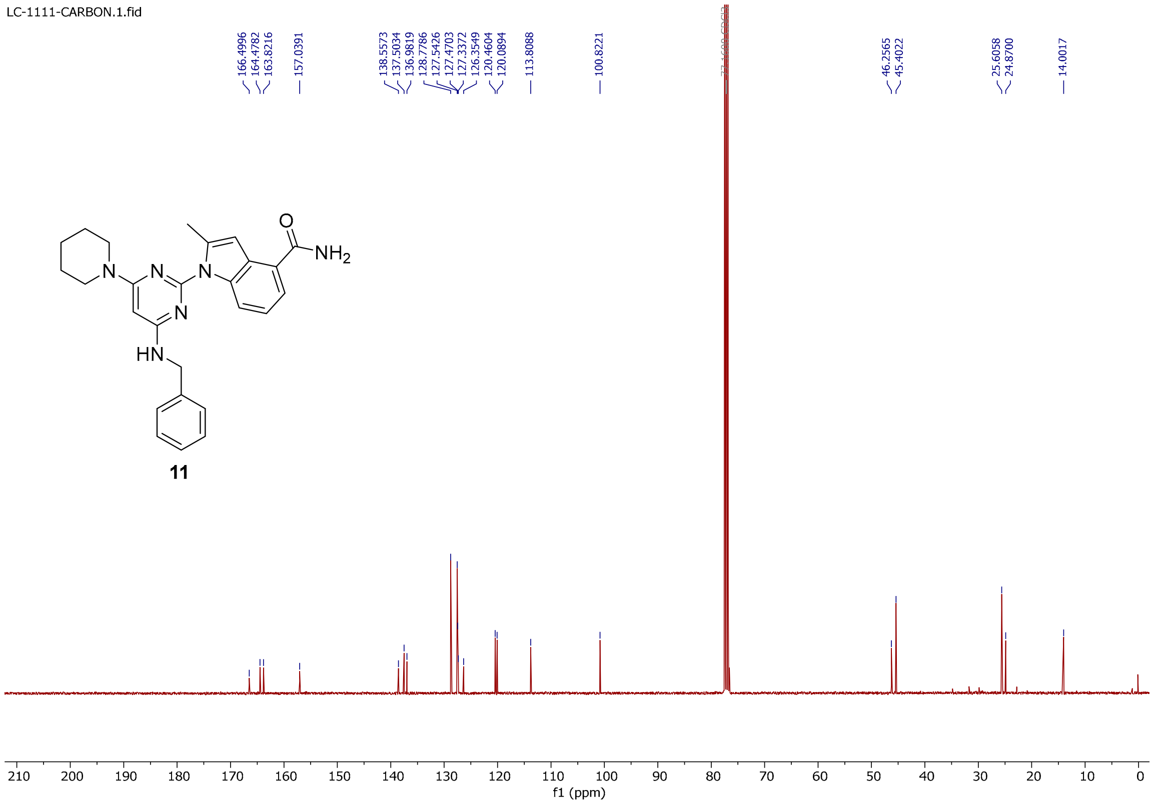

^{1}H and ^{13}C NMR spectra of compound **11**

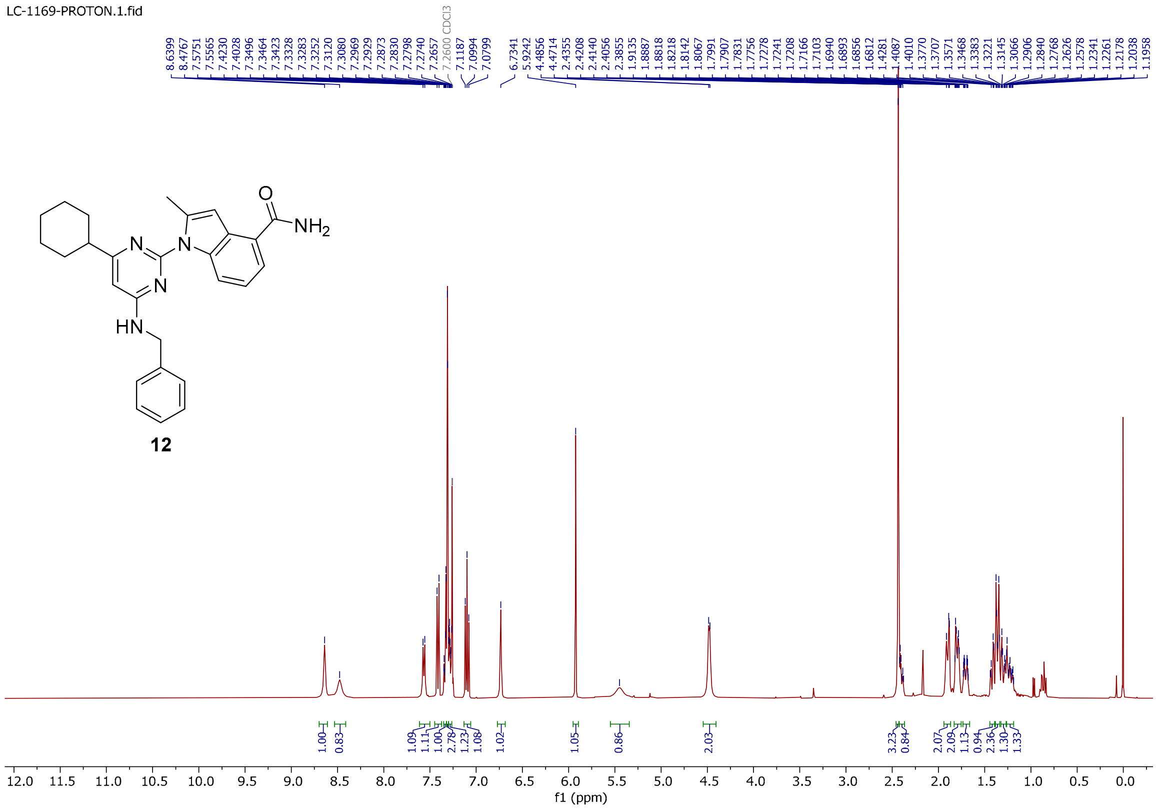

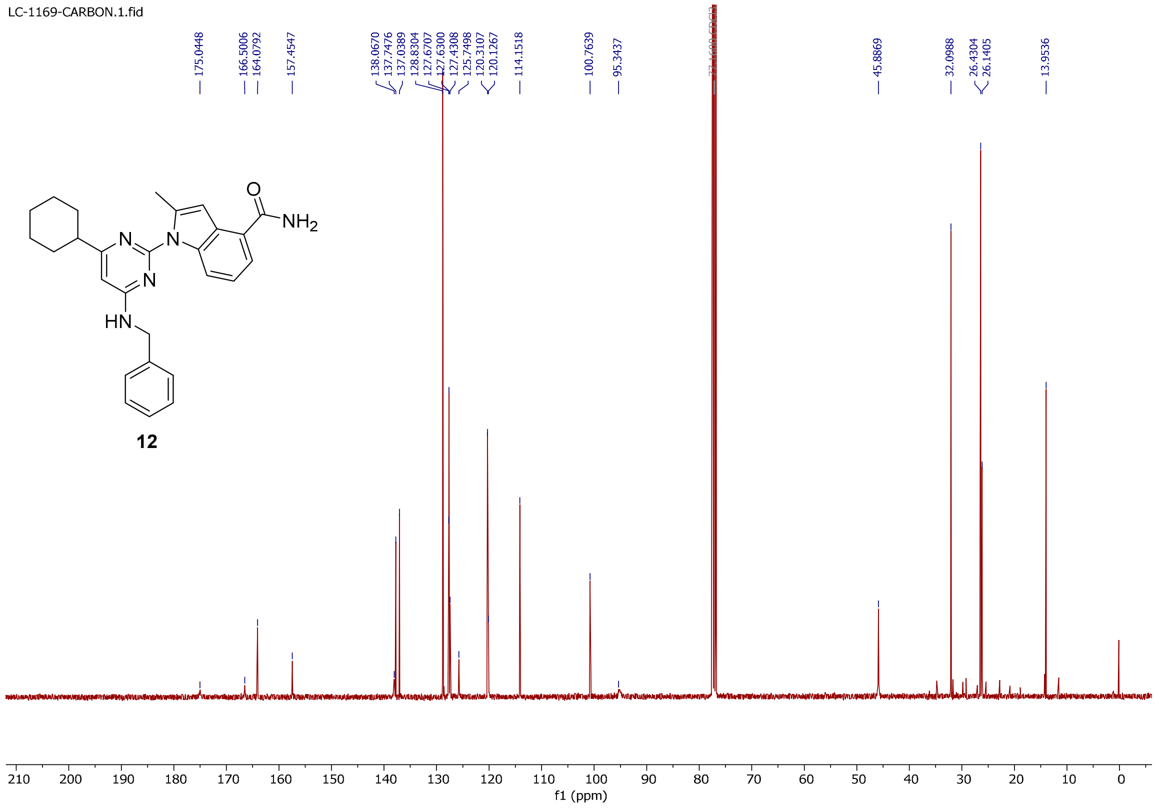

^{1}H and ^{13}C NMR spectra of compound **12**

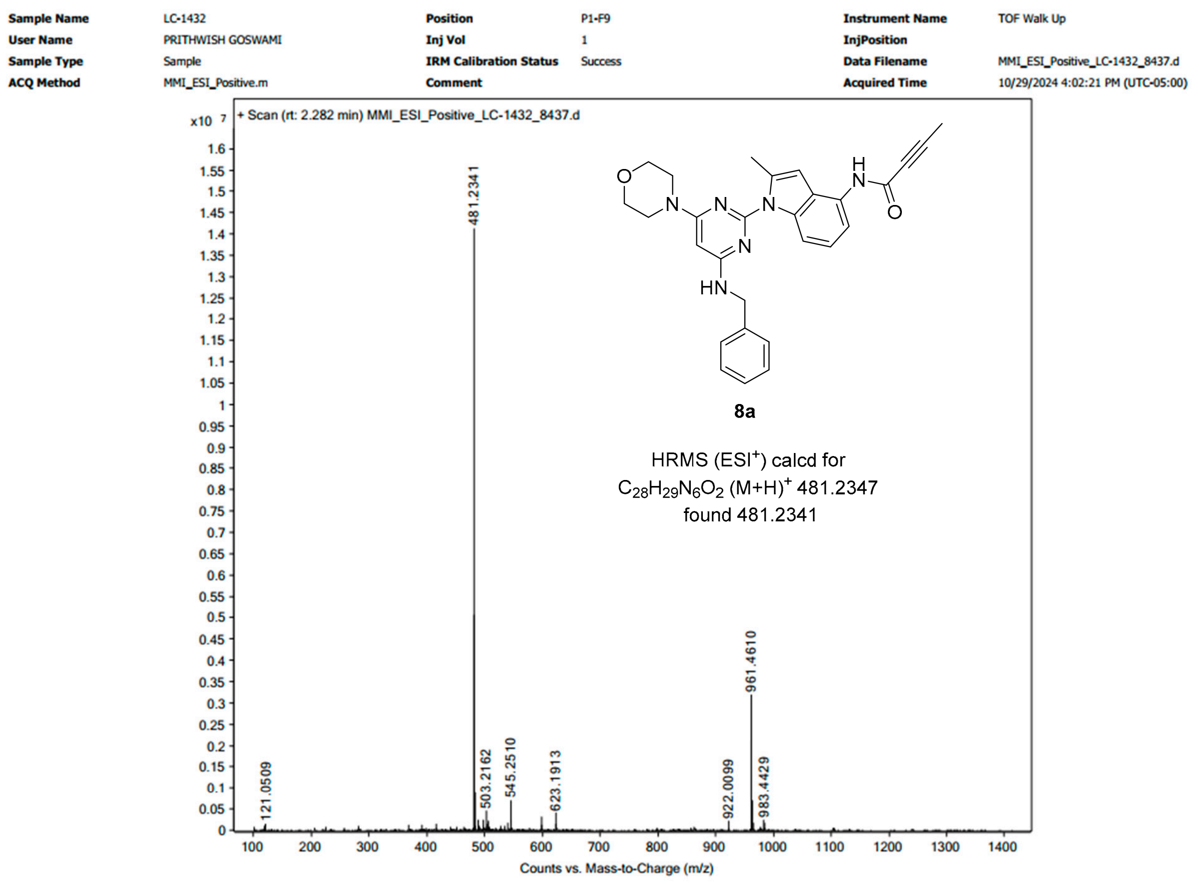

HRMS spectrum of compound 8a

**Sample Name**	LC-1500	**Position**	P2-A8	**Instrument Name**	TOF Walk Up
**User Name**	PRITHWISH GOSWAMI	**Inj Vol**	1	**InjPosition**	
**Sample Type**	Sample	**IRM Calibration Status**	Success	**Data Filename**	MMI_ESI_Positive_LC-1500_8469.d
**ACQ Method**	MMI_ESI_Positive.m	**Comment**		**Acquired Time**	10/30/2024 1:15:30 PM (UTC-05:00)

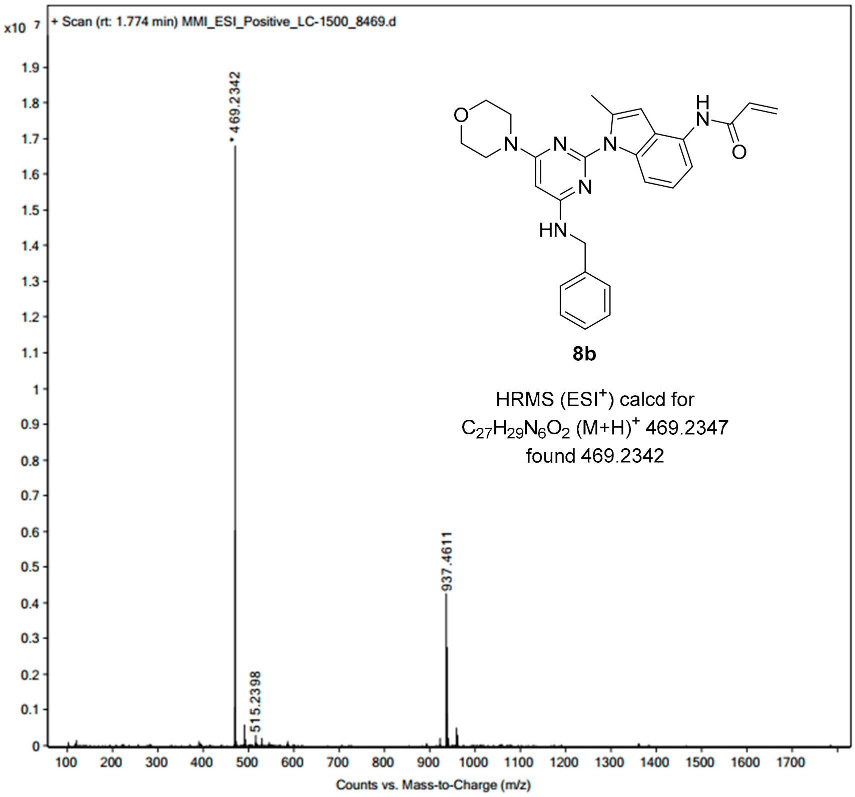

HRMS spectrum of compound 8b

**Sample Name**	LC-1503	**Position**	P2-A3	**Instrument Name**	TOF Walk Up
**User Name**	PRITHWISH GOSWAMI	**Inj Vol**	1	**InjPosition**	
**Sample Type**	Sample	**IRM Calibration Status**	Success	**Data Filename**	MMI_ESI_Positive_LC-1503_8453.d
**ACQ Method**	MMI_ESI_Positive.m	**Comment**		**Acquired Time**	10/29/2024 5:36:41 PM (UTC-05:00)

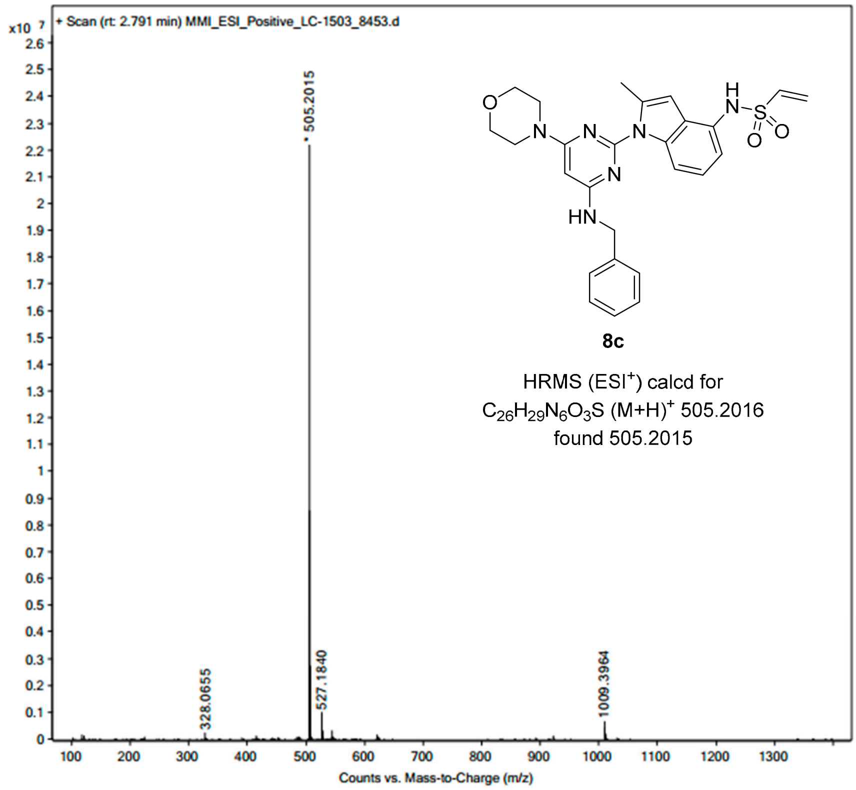

HRMS spectrum of compound 8c

**Sample Name**	LC-1433	**Position**	P2-B3	**Instrument Name**	TOF Walk Up
**User Name**	PRITHWISH GOSWAMI	**Inj Vol**	1	**InjPosition**	
**Sample Type**	Sample	**IRM Calibration Status**	Success	**Data Filename**	MMI_APCI_Negative_LC-1433_8492.d
**ACQ Method**	MMI_APCI_Negative.m	**Comment**		**Acquired Time**	10/30/2024 5:44:18 PM (UTC-05:00)

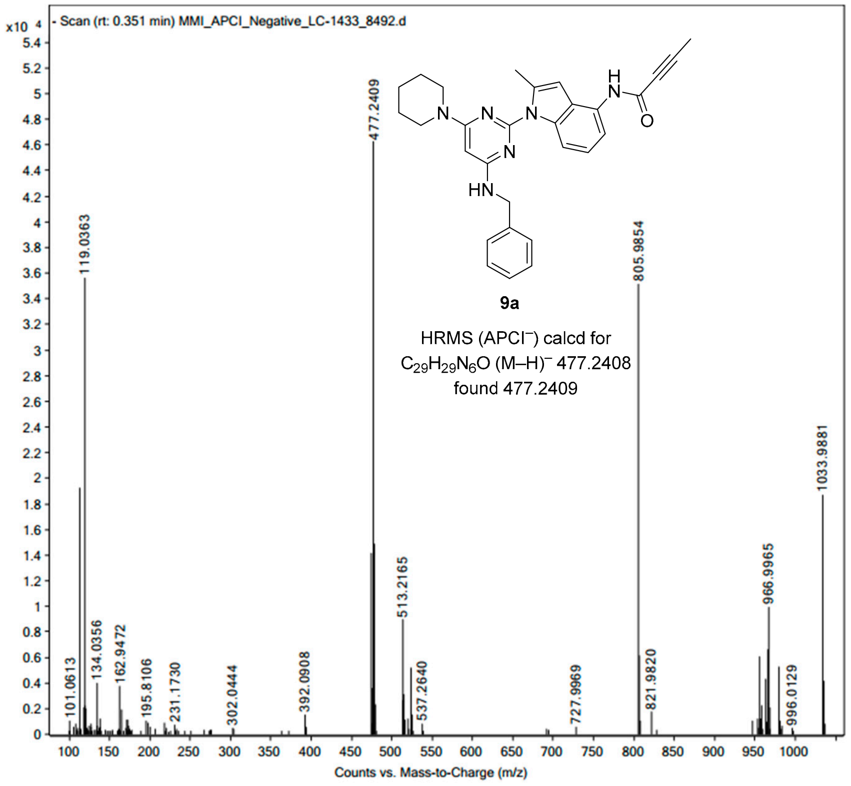

HRMS spectrum of compound 9a

**Sample Name**	LC-1499	**Position**	P2-B4	**Instrument Name**	TOF Walk Up
**User Name**	PRITHWISH GOSWAMI	**Inj Vol**	1	**InjPosition**	
**Sample Type**	Sample	**IRM Calibration Status**	Success	**Data Filename**	MMI_APCI_Negative_LC-1499_8496.d
**ACQ Method**	MMI_APCI_Negative.m	**Comment**		**Acquired Time**	10/30/2024 6:07:48 PM (UTC-05:00)

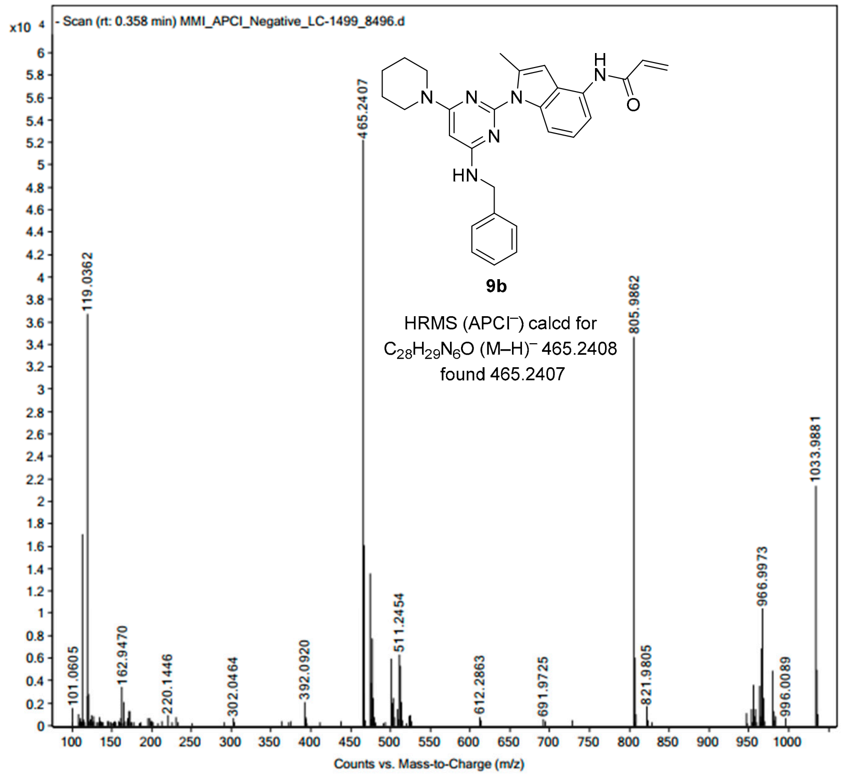

HRMS spectrum of compound 9b

**Sample Name**	LC-1502	**Position**	P2-B8	**Instrument Name**	TOF Walk Up
**User Name**	PRITHWISH GOSWAMI	**Inj Vol**	1	**InjPosition**	
**Sample Type**	Sample	**IRM Calibration Status**	Success	**Data Filename**	MMI_APCI_Negative_LC-1502_8509.d
**ACQ Method**	MMI_APCI_Negative.m	**Comment**		**Acquired Time**	10/31/2024 1:34:37 PM (UTC-05:00)

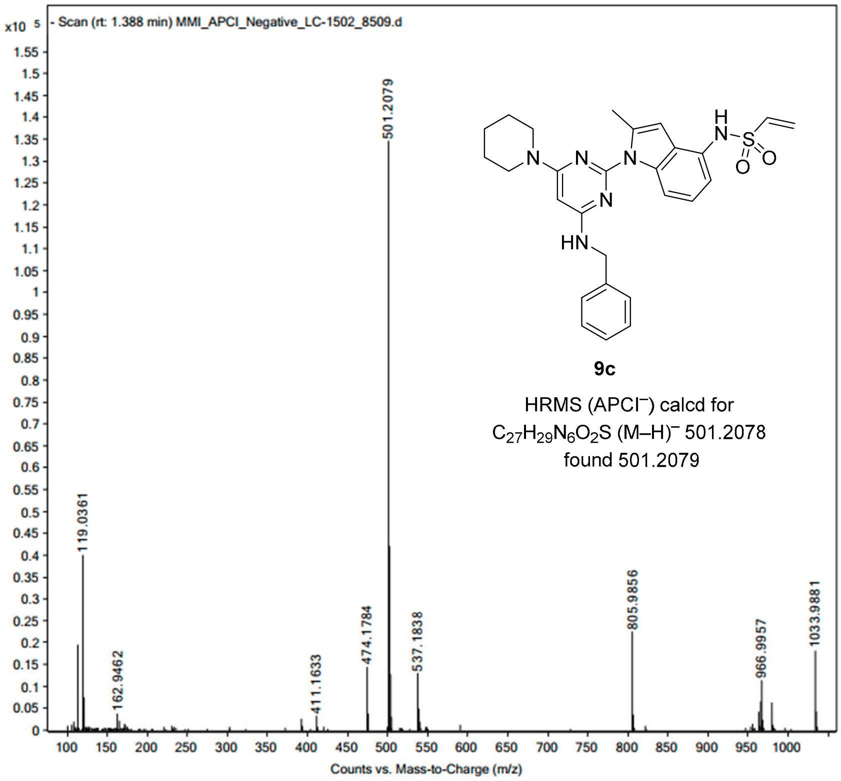

HRMS spectrum of compound 9c

**Sample Name**	LC-1046	**Position**	P2-E4	**Instrument Name**	TOF Walk Up
**User Name**	PRITHWISH GOSWAMI	**Inj Vol**	1	**InjPosition**	
**Sample Type**	Sample	**IRM Calibration Status**	Success	**Data Filename**	MMI_APCI_Negative_LC-1046_8601.d
**ACQ Method**	MMI_APCI_Negative.m	**Comment**		**Acquired Time**	11/1/2024 3:36:38 PM (UTC-05:00)

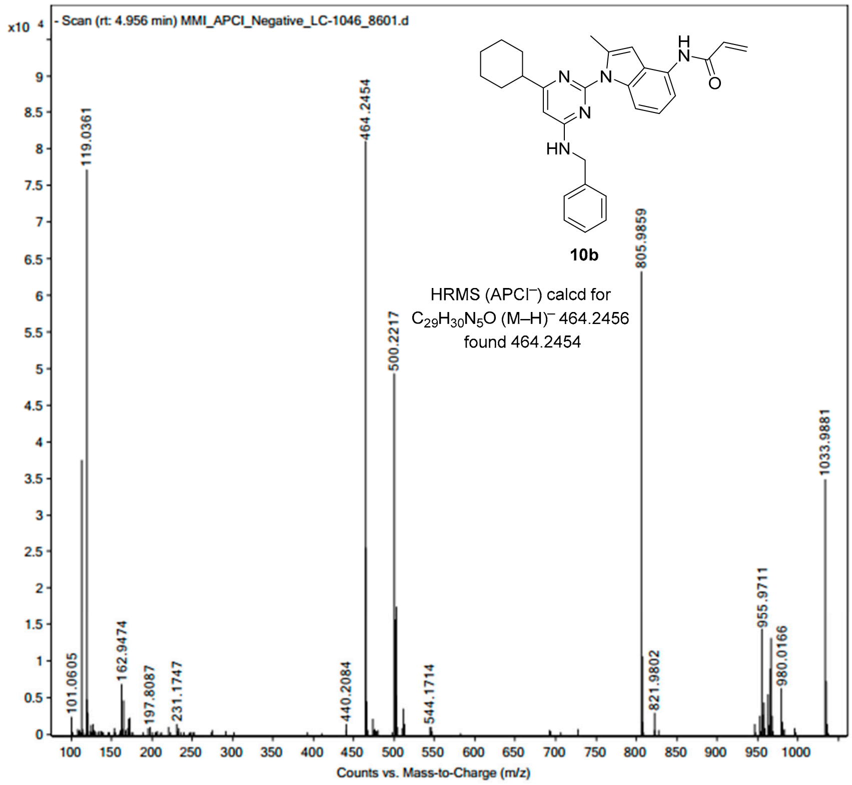

HRMS spectrum of compound 10b

**Sample Name**	LC-1501	**Position**	P2-C4	**Instrument Name**	TOF Walk Up
**User Name**	PRITHWISH GOSWAMI	**Inj Vol**	1	**InjPosition**	
**Sample Type**	Sample	**IRM Calibration Status**	Success	**Data Filename**	MMI_APCI_Negative_LC-1501_8526.d
**ACQ Method**	MMI_APCI_Negative.m	**Comment**		**Acquired Time**	10/31/2024 5:24:50 PM (UTC-05:00)

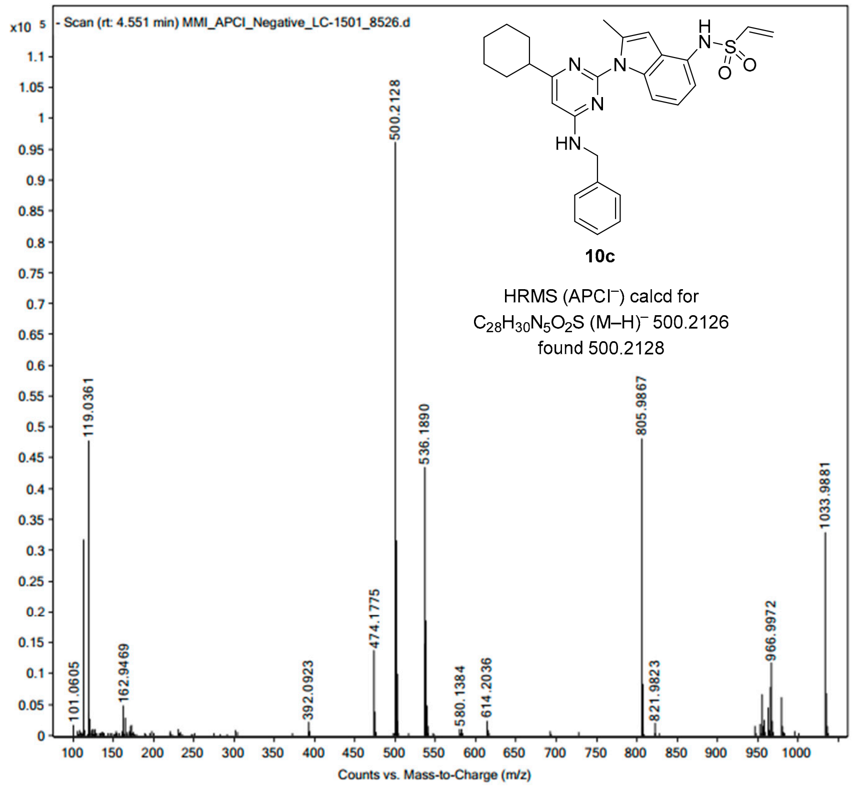

HRMS spectrum of compound 10c

**Sample Name**	LC-1111	**Position**	P1-B1	**Instrument Name**	TOF Walk Up
**User Name**	PRITHWISH GOSWAMI	**Inj Vol**	1	**InjPosition**	
**Sample Type**	Sample	**IRM Calibration Status**	Success	**Data Filename**	MMI_ESI_Negative_LC-1111_8718.d
**ACQ Method**	MMI_ESI_Negative.m	**Comment**		**Acquired Time**	11/5/2024 12:56:13 PM (UTC-06:00)

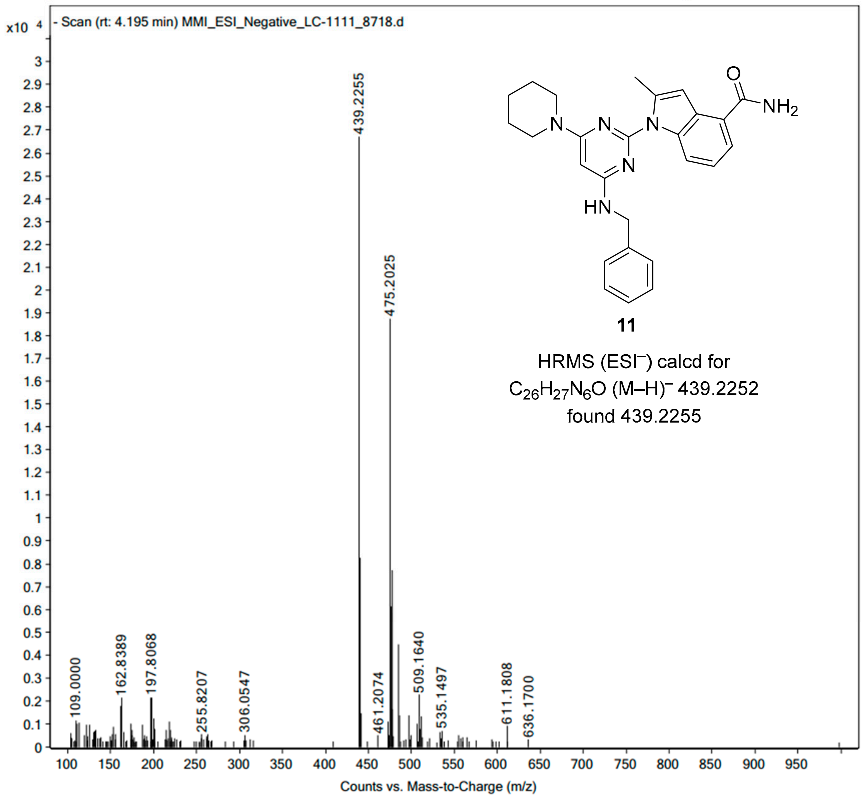

HRMS spectrum of compound 11

**Sample Name**	LC-1169	**Position**	P1-B7	**Instrument Name**	TOF Walk Up
**User Name**	PRITHWISH GOSWAMI	**Inj Vol**	1	**InjPosition**	
**Sample Type**	Sample	**IRM Calibration Status**	Success	**Data Filename**	MMI_ESI_Positive_LC-1169_8734.d
**ACQ Method**	MMI_ESI_Positive.m	**Comment**		**Acquired Time**	11/5/2024 2:30:39 PM (UTC-06:00)

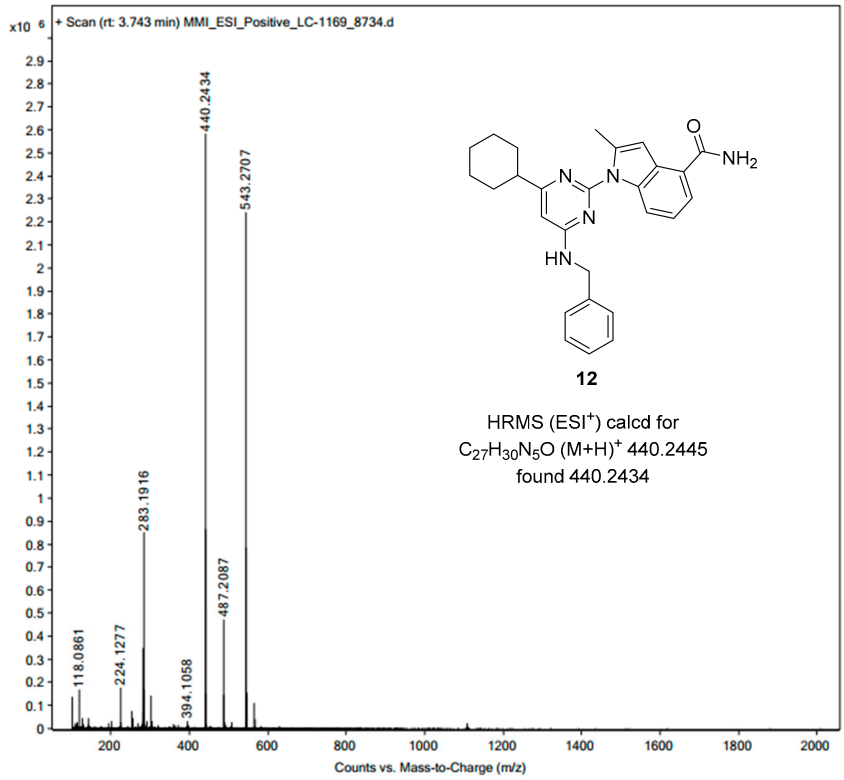

HRMS spectrum of compound 12
